# Implementing neurodevelopmental follow‐up care for children with congenital heart disease: A scoping review with evidence mapping

**DOI:** 10.1111/dmcn.15698

**Published:** 2023-07-08

**Authors:** Bridget R. Abell, Karen Eagleson, Benjamin Auld, Samudragupta Bora, Robert Justo, William Parsonage, Pakhi Sharma, Sanjeewa Kularatna, Steven M. McPhail

**Affiliations:** ^1^ Australian Centre for Health Services Innovation and Centre for Healthcare Transformation, School of Public Health and Social Work, Faculty of Health Queensland University of Technology Brisbane QLD Australia; ^2^ Queensland Paediatric Cardiac Service Queensland Children's Hospital Brisbane QLD Australia; ^3^ Faculty of Medicine The University of Queensland Brisbane QLD Australia; ^4^ University Hospitals Rainbow Babies & Children's Hospital Case Western Reserve University School of Medicine Cleveland OH USA; ^5^ Royal Brisbane and Women's Hospital Metro North Health Brisbane QLD Australia; ^6^ Digital Health and Informatics Directorate, Metro South Health Brisbane QLD Australia

## Abstract

**Aim:**

To identify and map evidence describing components of neurodevelopmental follow‐up care for children with congenital heart disease (CHD).

**Method:**

This was a scoping review of studies reporting components of neurodevelopmental follow‐up programmes/pathways for children with CHD. Eligible publications were identified through database searches, citation tracking, and expert recommendations. Two independent reviewers screened studies and extracted data. An evidence matrix was developed to visualize common characteristics of care pathways. Qualitative content analysis identified implementation barriers and enablers.

**Results:**

The review included 33 studies. Twenty‐one described individual care pathways across the USA (*n* = 14), Canada (*n* = 4), Australia (*n* = 2), and France (*n* = 1). The remainder reported surveys of clinical practice across multiple geographical regions. While heterogeneity in care existed across studies, common attributes included enrolment of children at high‐risk of neurodevelopmental delay; centralized clinics in children's hospitals; referral before discharge; periodic follow‐up at fixed ages; standardized developmental assessment; and involvement of multidisciplinary teams. Implementation barriers included service cost/resourcing, patient burden, and lack of knowledge/awareness. Multi‐level stakeholder engagement and integration with other services were key drivers of success.

**Interpretation:**

Defining components of effective neurodevelopmental follow‐up programmes and care pathways, along with enhancing and expanding guideline‐based care across regions and into new contexts, should continue to be priorities.

**What this paper adds:**

- Twenty‐two different neurodevelopmental follow‐up care pathways/programmes were published, originating from four countries.
- Twelve additional publications described broad practices for neurodevelopmental follow‐up across regions
- Common attributes across eligibility, service structure, assessment processes, and care providers were noted.
- Studies reported programme acceptability, uptake, cost, and effectiveness.
- Implementation barriers included service cost/resourcing, patient burden, and lack of knowledge/awareness.

## What this paper adds

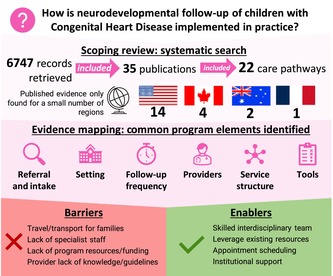

**Abbreviations**
AHA: American Heart Association
CHD: congenital heart disease

Advances in diagnosing and treating congenital heart disease (CHD) have resulted in more than 90% of children born with complex CHD now surviving into adulthood.^1^ Consequently, there has been an increased focus on assessing and understanding long‐term morbidity in this population including cognitive, learning, motor, and psychosocial impairments characterized as developmental delays and associated disabilities.^2^ Children requiring intervention for CHD are at an increased risk of experiencing such adverse developmental outcomes compared with children in the general population.^3^, ^4^, ^5^ While some impairments may be mild or improve over time, for a proportion of children, particularly those with single‐ventricle CHD, neurodevelopmental challenges can be severe, cumulative, or persist across the lifespan.^3^, ^5^, ^6^ Adverse impacts have been observed on education, employment, social interactions, functional independence, and quality of life.^3^ This also has implications for health and social services, with an increasing number of children with CHD requiring access to psychological, educational, and behavioural support services.^6^, ^7^, ^8^ Consequently, screening and early identification of delays and deficits, along with continuing surveillance of children with CHD, are important to facilitate early intervention, and plan for resource allocation for this population.

The American Heart Association (AHA) has proposed a three‐layered model of increasing intensity for the provision of neurodevelopmental follow‐up care for children with CHD.^1^ In the first instance, routine developmental surveillance or monitoring should be provided to all children with CHD by their primary care provider. A more comprehensive follow‐up approach of developmental screening with standardized tests or scales is recommended for children with CHD who are identified as being at a greater risk of adverse developmental outcomes (because of their diagnosis or other concerns). Finally, specialized evaluation can be performed by trained professionals to provide a developmental or behavioural diagnosis, and enable access to intervention services, particularly for children identified as high risk. Since the publication of these AHA recommendations, the provision of neurodevelopmental follow‐up programmes has increased as a way of supporting both structured screening and evaluation of this population.^9^, ^10^

An overarching purpose of neurodevelopmental follow‐up programmes is to provide comprehensive developmental care to children with CHD by implementing evidence‐based recommendations for clinical practice.^11^ These programmes have traditionally been run centrally by hospitals providing surgical interventions for CHD. They have typically included multidisciplinary teams of providers, with elements including care coordination, neurodevelopmental assessment, provision of education and resources, and referral to therapies. Despite the increasing role these programmes play in providing long‐term neurodevelopmental follow‐up for children with CHD, there has been a paucity of research that has considered programme design or effectiveness.^10^ Moreover, no recent research has built on early attempts to synthesize common programme approaches and implementation challenges.^12^ Surveys of current practice have also highlighted considerable variability in the delivery of neurodevelopmental follow‐up care both within and across countries.^13^, ^14^, ^15^, ^16^ Importantly, understanding different programme approaches and pathways of care that can be adopted is critical to guide context‐relevant implementation, inform scale‐up, and perform comparative evaluation of their effectiveness in practice.^10^

Consequently, we aimed to perform a scoping review to systematically identify and map evidence describing components of neurodevelopmental follow‐up care for children with CHD. This included categorizing the described programmes, pathways, and outcomes and highlighting contextual barriers and enablers affecting implementation. A scoping review approach aligned best with this aim to identify and map key neurodevelopmental follow‐up approaches, implementation characteristics, identify knowledge gaps, and to explore how research has been conducted in the field rather than evaluate this evidence.^17^

## METHOD

This scoping review was conducted systematically in accordance with the recommendations of Arksey and O'Malley^18^ and Levac et al.^19^ Aligned with this guidance, the core multidisciplinary review team (BRA, BA, KE, SB, SMM) who were experienced in paediatric cardiology (medical and nursing), psychology, physiotherapy, and health services research met regularly to discuss issues related to review scope, study selection, data extraction, and data presentation. Before starting the review, the research team developed a protocol using the abovementioned guidance but this was not registered or published (Appendix S1). We used The Preferred Reporting Items for Systematic Reviews and Meta‐Analyses extension for Scoping Reviews (PRISMA‐ScR) checklist^20^ to guide reporting of this scoping review.

### Stage 1: research question

The research question asks: what programmes and care pathways are reported in the international literature for the neurodevelopmental follow‐up of children with CHD? We defined a care pathway as a multicomponent intervention for the organization of care processes for a well‐defined group of patients (children with CHD) during a well‐defined period.^21^ In this case, the period of interest was between the ages of 0 and 18 years, before they were transitioned to adult care services.^22^ Follow‐up care processes could include surveillance, screening, or evaluation of the population.

### Stage 2: identifying relevant studies

The search strategy was iteratively created by combining MeSH (Medical Subject Headings) terms and keywords related to the population (children, congenital heart disease) and concept (neurodevelopmental follow‐up) of the review. Using published reviews in related fields and the knowledge of subject matter experts, a search string was used to perform a preliminary search of Ovid MEDLINE (Table S1). This string was subsequently refined with the SearchRefinery tool^23^ by removing low‐value/high‐noise terms and retaining key terms using PubMed reference numbers of eight relevant articles returned through the original search and a complementary Google Scholar search.

The final search terms are provided in Table 1. The strategy was used to search four bibliographic databases from inception to August 2021: Ovid MEDLINE, Embase, CINAHL, and Scopus. The Polyglot Search Translator^24^ was used to translate the Ovid MEDLINE string across all other databases (Appendix S2). The final search results were uploaded into EndNote (version X9; Clarivate, Philadelphia, PA, USA), duplicates removed, and remaining records uploaded into EPPI‐Reviewer^25^ for screening. Generation of the search terms, execution of all searches, and deduplication of records were all led by the same author (BRA) in consultation with a medical librarian. The electronic database search was supplemented by additional publications identified by subject matter experts in the team, as well as forward citation searching of included records. An additional search using the same methods was conducted in March 2023 owing to the time elapsed between the initial search and completion of the review.

**TABLE 1:** Scoping review search terms.

Population (children)	Population (congenital heart disease)	Concept (neurodevelopmental follow‐up)
exp Child/ OR Adolescent/ OR child*.tw. OR infant*.tw. OR famil*.tw. OR ?school.tw. OR p?ediatr*.tw. OR prematur*.tw.	Heart Defects, Congenital/ OR ‘congenital heart disease*’.tw. OR CHD.tw. OR congenital heart.tw. OR (heart ADJ1 surgery).tw	exp Neurodevelopmental Disorders/ OR Child Development/ OR ‘Referral and Consultation’/ OR ‘Delivery of Health Care’/ OR Cognition Disorders/ OR Language Disorders/ OR Psychomotor Disorders/ OR ‘early intervention’.tw. OR neurodevelopment*.tw.)

### Stage 3: study selection

#### Eligibility criteria

Inclusion and exclusion criteria were decided at the start of the scoping review and refined throughout, as described in the protocol (Appendix S1). There were only two inclusion criteria: (1) the population of focus in the study was infants, children, or young people (0–18 years old) who had been diagnosed with CHD (population); and (2) the study described components of a model of care, process, pathway, or programme for neurodevelopmental or developmental follow‐up, screening, surveillance, or evaluation of the population of interest and/or their families (concept/phenomena of interest). Definitions of surveillance, screening, and evaluation were adopted from the current AHA Scientific Statement for evaluation and management of neurodevelopmental outcomes in children with CHD.^2^

Original research studies, systematic/scoping reviews, and conference abstracts of any study design (e.g. randomized controlled trial, cohort, case study, observational, cross‐sectional, quasi‐experimental) published in a peer‐reviewed journal were eligible for inclusion. These could include qualitative, quantitative, and mixed‐methods approaches. Publications meeting the inclusion criteria were not excluded on the basis of language, publication date, or geographical region.

Review exclusion criteria were the following: (1) studies focused on children with acquired heart disease (different population); (2) studies focused on adults with CHD (different population); (3) studies about the transition from paediatric to adult follow‐up of CHD (different concept/context); (4) studies that reported a model of care, process, pathway, or programme for neurodevelopmental/developmental care in inpatient neonatal, paediatric, or cardiac intensive care units without continuing follow‐up after hospital discharge (different concept/context); (5) publications that reported a model of care, process, pathway, or programme for neurodevelopmental or developmental follow‐up, screening, surveillance, or assessment but did not describe at least two individual components of this model, for example provider, assessment tools, setting, patient eligibility (lack of concept/context); (6) studies where developmental support was provided wholly outside the health‐care sector, including by education and disability providers (different context); (7) published abstracts without adequate detail to determine eligibility and no subsequent full‐text publication available or information from authors to assist in screening; (8) narrative reviews, commentaries, editorials, viewpoints, or guidelines/policy statements.

#### Screening

Preliminary screening was piloted by all reviewers with a small sample of records to improve consistency of study selection criteria. The titles and abstracts of all records were independently co‐screened in EPPI‐Reviewer by four reviewers (BRA, BA, KE, NH) working in pairs. Any discrepancies were discussed among the group by considering study inclusion and exclusion criteria until an agreement was reached. The same reviewers then also independently co‐screened the full‐text publications using the same methods to determine study inclusion (agreement by two reviewers) and resolve any disagreements.

### Stage 4: charting the data

A data extraction spreadsheet was developed by one reviewer (BRA), and piloted and refined by three members of the review team (BRA, KE, PS) using several included studies. Data from all included studies were extracted in Microsoft Excel using this standardized template. Two reviewers (BRA and PS) charted data from the remaining eligible publications, with data from 10% of records being verified for accuracy and completeness by an additional reviewer (KE).

Data items captured included publication details (author, year, journal, publication type, conference details, country); study details (aim/focus, design, dates of data collection, data collection methods, outcomes reported, data analysis methods); programme or pathway details (clinic/programme name, health‐care setting, geographical location, date of establishment, service structure, eligibility criteria, providers, follow‐up scheduling, referral pathways and processes, follow‐up domains and processes, standardized tools used, carer or family involvement, intervention/therapy provided, service partnerships, description of electronic database/registry); child/participant details (number, age, diagnosis, clinical role); determinants of implementation (barriers to implementation of programme/pathway, enablers to implementation of programme/pathway); implementation outcomes (types such as acceptability, how they were measured, reported results); and service outcomes (types such as access/effectiveness and cost, how they were measured, reported outcomes). These items could be described in the methods, results, or discussion sections of included publications.

### Stage 5: collating and summarizing results

For ease of interpretation, we grouped studies by individual programmes/care pathways and surveys of national/international practice. Summaries of the characteristics of care pathways for each of these groups of publications were reported in separate tables but reporting of implementation determinants (barriers and enablers) encompassed both sets of studies. In all summary tables we grouped publications by the country in which the follow‐up programme/care pathway was located, or where the survey was performed. An evidence matrix was developed to categorize and visualize information about shared and unique characteristics of care pathways across individual programmes/publications. Our approach to identifying implementation determinants involved basic qualitative content analysis of all included publications (qualitative, quantitative, and mixed methods) using open coding and subsequent allocation into higher‐order categories.^26^ This was performed by a review member skilled in qualitative analysis (BRA).

## RESULTS

The searches returned 6747 potentially relevant publications, with 5426 remaining for title and abstract screening after deduplication. From these, 189 full‐text papers were sought and screened for eligibility. An additional three publications were identified for full‐text review by subject matter experts and one from forward citation searching of conference abstracts. Full‐text screening of these 193 publications resulted in 35 publications being included in this review.^9^, ^13^, ^14^, ^15^, ^16^, ^27^, ^28^, ^29^, ^30^, ^31^, ^32^, ^33^, ^34^, ^35^, ^36^, ^37^, ^38^, ^39^, ^40^, ^41^, ^42^, ^43^, ^44^, ^45^, ^46^, ^47^, ^48^, ^49^, ^50^, ^51^, ^52^, ^53^, ^54^, ^55^, ^56^ The stages of the search and screening process and reasons for exclusion are presented in the PRISMA‐ScR flow diagram (Figure S1).

### Characteristics of included studies

The 35 publications included in the scoping review comprised 33 different studies. Of these, 21 studies examined the implementation of specific programmes or care pathways for neurodevelopmental follow‐up care,^27^, ^28^, ^29^, ^30^, ^31^, ^32^, ^33^, ^34^, ^35^, ^36^, ^37^, ^38^, ^39^, ^40^, ^42^, ^44^, ^47^, ^48^, ^49^, ^50^, ^51^, ^52^, ^53^, ^54^, ^56^ and 12 studies reported on surveys or overviews of current clinical practices in neurodevelopmental follow‐up.^9^, ^13^, ^14^, ^15^, ^16^, ^30^, ^37^, ^41^, ^43^, ^45^, ^46^, ^55^

#### Implementation and evaluation of follow‐up programmes or care pathways

All studies were published after 2011, with most (15 out of 21) conducted since 2018. Fourteen of the studies reported on neurodevelopmental care pathways or programmes based in the USA,^27^, ^32^, ^33^, ^34^, ^35^, ^36^, ^38^, ^39^, ^40^, ^42^, ^44^, ^46^, ^47^, ^48^, ^49^ with four in Canada,^50^, ^51^, ^52^, ^56^ two in Australia,^31^, ^53^, ^54^ and one in Europe.^28^, ^29^ All were observational in design, with only four studies using comparator groups.^32^, ^35^, ^39^, ^51^ Most studies were small, involving fewer than 100 children. However, one study reported on outcomes for more than 650 children over a 15‐year period.^50^ Two other studies^33^, ^56^ reported that their programmes had enrolled large numbers of children although only reported on outcomes for a much smaller subset of these children in the included publications. In total, 3132 children with a wide range of congenital heart conditions were included as participants across the 20 studies. Most studies (15 out of 21) reported on the effectiveness of their programme or care pathway for improving access to neurodevelopmental care or diagnoses for children with CHD. Fewer studies reported on implementation barriers, and associated outcomes such as acceptability, adoption, and cost. A summary of study characteristics for this set of publications is presented in Table S2.

#### Surveys and overviews of neurodevelopmental follow‐up practices

Papers were published between 2016 and 2022, reporting on neurodevelopmental follow‐up practices across five international regions. All but two were prospective surveys sent to providers of care for children with CHD based in hospitals, academic centres, community clinics, cardiac clinics, and private practices. Individual participants in the surveys worked in a variety of roles; however, cardiologists, psychologists, paediatricians, and nurses were best represented across all surveys. In total 194 sites, plus 455 individuals, were surveyed across these 10 studies. Some of the sites may have participated in more than one of the included surveys. The remaining two papers included (1) an analysis of neurodevelopmental follow‐up care for a cross‐sectional sample of children with CHD in the UK^43^ and (2) description of the implementation of a national registry and standardized neurodevelopmental follow‐up care across Switzerland.^30^ A summary of study characteristics for this set of publications is presented in Table S3.

### Review findings

The following summary of findings is separated into characteristics observed in individual neurodevelopmental programmes or care pathways and those seen more broadly in surveys and overviews of clinical practice.

#### Characteristics of individual care pathways for neurodevelopmental follow‐up

**FIGURE 1:**
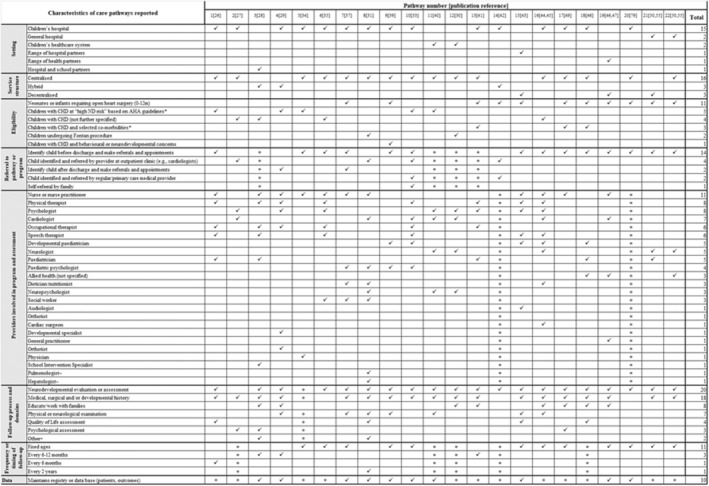
Evidence map of reported characteristics of care pathways for neurodevelopmental follow‐up of children with congenital heart disease. ✓, characteristic of care pathway reported in publication; •, publication did not report on this care pathway characteristic; *, as categorised in 2; ~, as part of larger follow‐up program of care not specific to neurodevelopment; + other processes included school education/advocacy, developing CHD action plans, developing and sharing education plans and recommendations, and conducting bloods tests, imaging, and cardiopulmonary testing.

The 21 studies that described neurodevelopmental follow‐up at individual sites came from 17 different hospitals or clinical centres and reported 22 different neurodevelopmental follow‐up care pathways. Some centres had multiple publications about the same care pathway (e.g. Clinique d'Investigation Neurocardiaque^51^, ^52^) while other centres had multiple publications reflecting each of the care pathways available in their service (e.g. Herma Heart Centre^27^, ^32^, ^33^). A visual representation of the relationship between publications, clinical centres, and care pathways is available in Figure S2. Detailed descriptions of each neurodevelopmental care pathway are provided in Table S4. The common and unique elements of care pathways are provided in an evidence map (Figure 1).

##### Follow‐up location, service structure, and providers

Most care pathways (14 out of 22) were established between 2010 and 2015 and located in the USA (14 out of 22). They were most frequently found in children's hospitals (15 out of 22), with four provided across multiple health‐care settings.^35^, ^47^, ^50^, ^53^ Almost three‐quarters (16 out of 22) had a centralized service structure, with families required to travel to a specific tertiary‐level clinic to receive care. Four were largely centralized but had elements of flexibility including outreach into schools,^33^ an initial telephone‐based developmental assessment,^34^ and assessment and follow‐up by providers closer to home for some children.^28^, ^29^, ^49^ Only three care pathways were provided in a completely decentralized manner across a catchment region^29^, ^50^, ^53^ using locally based neonatal and follow‐up clinics, community‐based supports, parental‐led questionnaires, primary care physicians, or primary care child health services to provide follow‐up and evaluation. Neurodevelopmental follow‐up was delivered by multidisciplinary clinical teams in all care pathways. More than 20 different types of provider were reported as taking part in included programmes with psychologists/paediatric psychologists (*n* = 12), nurses/nurse practitioners (*n* = 11), physical therapists (*n* = 8), cardiologists (*n* = 7), speech therapists (*n* = 6), and occupational therapists (*n* = 6) the most frequently reported clinicians involved in care. Ten programmes described using a database or registry to track patients and their outcomes.^31^, ^33^, ^34^, ^36^, ^38^, ^42^, ^44^, ^47^, ^50^, ^53^

###### Accessing follow‐up care

Eligibility criteria for programmes varied widely but most included children undergoing early interventions or surgery for CHD and/or who were classified at high risk of developmental disorder according to the AHA.^2^ The most common method of referral was to identify eligible children during their neonatal admission at the enrolling hospital and refer them to the programme or neurodevelopmental follow‐up pathway before discharge (*n* = 14). Hospital outpatient clinics (*n* = 4) were also key referral sources. Only one programme reported accepting self‐referrals.^38^

###### Follow‐up care processes

Most clinics (*n* = 11) saw patients periodically at fixed ages such as 6 months, 12 months, 18 months, and 4 years. However, several followed up children every 6 to 12 months^27^, ^34^, ^48^ or 2 years.^36^ The actual processes performed during each follow‐up visit were generally not well or consistently described across publications. However, the most common practices reported were taking a medical, surgical, and/or developmental history, and conducting neurodevelopmental screening or evaluation. Only four care pathways explicitly reported assessing children's health‐related quality of life,^32^, ^36^, ^50^, ^53^ and only three explicitly reported performing psychological assessment or consultation.^32^, ^33^, ^56^ Five care pathways reported providing families with feedback, education, and resources about the importance of neurodevelopmental follow‐up^33^, ^34^, ^48^, ^53^, ^54^, ^56^ and two reported offering psychological support for parents such as counselling.^35^, ^51^ Only one care pathway reported assessment of parental quality of life or mental health.^53^ Thirty‐two different tools were used in standardized assessments and evaluation of children across the 22 care pathways. However, only five of these tools were used across more than three individual pathways. These were Bayley Scales of Infant and Toddler Development (*n* = 11), Ages and Stages Questionnaire (*n* = 5), Child Behaviour Checklist (*n* = 5), and Behavior Assessment System for Children (*n* = 4). Health‐related quality of life was most frequently assessed using the Pediatric Quality of Life Inventory (*n* = 4).

#### Characteristics of neurodevelopmental follow‐up practices observed in surveys and overviews

Twelve publications described broad practices for neurodevelopmental follow‐up across selected sites and providers in the USA, Canada, Europe (Spain, France, the Netherlands, Germany, Switzerland, Hungary, Slovenia, Italy, Austria, Latvia, UK), and South Africa (see Table S5 for detailed descriptions). Although they provided a good overview of current programmes and services offered, details about the specific elements of programmes (such as eligibility criteria, referral pathways, assessment tools) were not well reported. Care pathways included routine surveillance by individual practitioners, referral to specialized providers for formal evaluation, psychological evaluation only, integration with neonatal follow‐up of all high‐risk infants, structured neurodevelopmental programmes, early intervention services, and care as usual (no formal process). Similar providers were involved in care as seen in the individual programmes with psychologists, paediatricians, nurses, and cardiologists reported as key contributors to assessment. The Bayley Scales of Infant and Toddler Development was again the most frequently reported assessment tool (*n* = 5), with a range of others also being regularly used. One survey additionally described the implementation of telehealth practices in structured neurodevelopmental follow‐up programmes during the COVID‐19 pandemic.^46^

The key finding across all surveys was that neurodevelopmental follow‐up practices are currently highly varied and perceived to be suboptimal both within and across geographical regions. There was also a lack of familiarity with the AHA neurodevelopmental follow‐up guidelines for children with CHD in a large proportion of the providers surveyed. Delivery of specialized cardiac neurodevelopmental follow‐up programmes varied in frequency and was lacking at up to one‐half of sites in some of the surveys conducted in the USA, Europe, and Canada. Additionally, in one survey of paediatricians and family physicians,^9^ only half routinely referred children with CHD for formal developmental evaluation. Similarly, in another survey,^55^ only 45% of paediatric cardiologists without access to a developmental follow‐up programme routinely performed neurodevelopmental surveillance. Consequently, authors of these surveys perceived care to be suboptimal, and concluded that many high‐risk children did not seem to be receiving appropriate care to meet their developmental needs. Conversely, recent surveys of institutional sites participating in the Cardiac Neurodevelopmental Outcome Collaborative^46^ and Children's Hospital's Neonatal Consortium^45^ demonstrated higher levels of service provision for these children (≥90% of sites performing neurodevelopmental assessment and/or evaluation).

Key recommendations from included survey studies were adaptation of AHA guidelines to local context, increasing standardized and systematic approaches, better collaboration between primary care providers and paediatric cardiologists, and improving management for school‐aged children.

#### Barriers and enablers to implementation of neurodevelopmental follow‐up care for children with CHD

**FIGURE 2:**
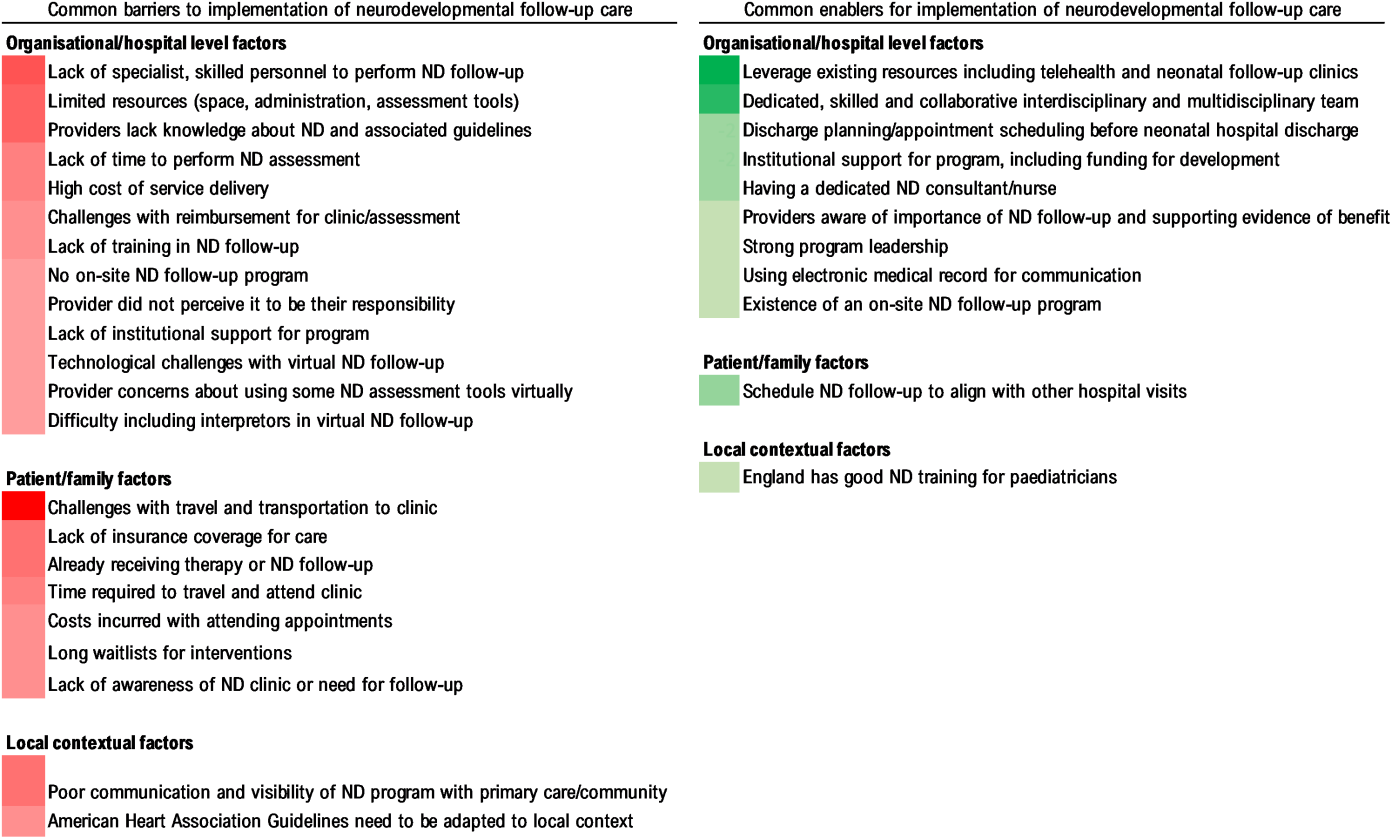
Factors influencing implementation and uptake of neurodevelopmental (ND) follow‐up practices. Darker colours represent a larger number of publications reporting that factor.

Fifteen studies^13^, ^14^, ^15^, ^27^, ^32^, ^34^, ^35^, ^36^, ^38^, ^40^, ^42^, ^43^, ^46^, ^53^, ^55^ identified one or more factors that influenced the implementation of neurodevelopmental follow‐up care practices for children with CHD. We classified these as either barriers or enablers to implementation at the level of the patient, organization/hospital, or local context (Figure 2).

Barriers were more commonly reported than enablers. The most frequently reported barrier for patients and families was travelling to centralized clinics for appointments (reported in 8 of the 15 studies). At the programme/organizational level, a limited workforce of specialist staff skilled in neurodevelopmental care was the most frequently reported barrier to delivering services (6 out of 15 studies). This was closely followed by limited access to other resources (space, administration staff, standardized tests), and providers lacking knowledge about neurodevelopmental follow‐up or the AHA guidelines (reported in five studies each). Financial issues (such as high programme costs, limited health‐care budgets, and out‐of‐pocket costs) also negatively affected programme implementation and uptake at the organizational and patient levels. Communication between hospital‐based providers and those in primary care community services was also an implementation barrier, particularly for service visibility and perceived roles and responsibilities for patient care. Two publications^15^, ^43^ highlighted the challenges associated with trying to apply a US‐developed guideline to different health‐care systems (UK and Canada). Another reported on the difficulties of implementing neurodevelopmental follow‐up practices through telehealth including provider concerns about delivering assessment virtually, technological issues for families and providers, and integration of interpreters.^46^

Overall, publications reported fewer common enabling factors than barriers. However, the importance of a dedicated and skilled team who worked collaboratively within and across disciplines to deliver neurodevelopmental follow‐up was a common finding in five studies. Leveraging existing resources within and beyond the organization to deliver follow‐up care (for example, local services, telehealth, neonatal follow‐up clinics, or clinical networks) was commonly perceived as a way of overcoming the reported challenges associated with staffing and resourcing (reported in six studies). Finally, the use of telehealth necessitated by the COVID‐19 pandemic also enabled increased reach and uptake of follow‐up services.

#### Outcomes of implementing neurodevelopmental follow‐up programmes and care pathways for children with CHD

Studies reported outcomes associated with both programme/care pathway implementation and subsequent effectiveness in providing access to additional evaluation, a new behavioural diagnosis, or referral to therapy. However, none of these were derived from randomized controlled designs, and only four studies used comparator groups to quantify the impact of their programme. Rather, most of the outcomes reported emerged from retrospective chart audits of patient cohorts attending single‐centre programmes, with some using a pre–post implementation design. While one study estimated it cost US$1000 per patient per year to add a school‐based liaison to their programme,^33^ no studies in our sample reported on overall programme costs or cost‐effectiveness.

Fifteen studies,^27^, ^28^, ^29^, ^31^, ^32^, ^33^, ^34^, ^36^, ^40^, ^42^, ^49^, ^50^, ^51^, ^53^, ^56^ reported outcomes related to neurodevelopmental programme adoption (uptake) by eligible children with CHD. The lowest reported uptake of neurodevelopmental follow‐up was 4%,^38^ accounted for by the low number of providers regularly referring into the programme, and patient no‐shows due to insurance coverage. This was, however, an outlier, with the median rate of uptake across all 15 studies high at 79%. Two Canadian programmes^50^, ^51^ reported adoption rates as high as 96% of all eligible children within their catchments. A newly established programme in France^29^ reported 100% adoption of parental screening with Ages and Stages Questionnaires through a general paediatrician neurodevelopmental follow‐up pathway.

Only six studies^27^, ^28^, ^29^, ^33^, ^34^, ^35^, ^53^ reported on the acceptability of their programme, using measures of carer satisfaction or continued clinic attendance as indicators. Three publications^33^, ^34^, ^53^ simply reported that feedback and surveys of families were ‘positive’ and had demonstrated ‘high levels of parent satisfaction’ with the care pathway provided by their clinic. The only two studies to provide direct measurement of acceptability from a programme survey reported 96.5%^35^ and 94%^28^ of parents to be satisfied with the neurodevelopmental evaluation process. The final study used its high rate of return clinic visits (85%) as a proxy indicator to demonstrate satisfaction.

Most studies (15 out of 21)^32^, ^33^, ^34^, ^35^, ^36^, ^39^, ^44^, ^47^, ^48^, ^49^, ^50^, ^51^, ^52^, ^54^, ^56^ reported on the impact of the neurodevelopmental follow‐up programme/pathway on providing new diagnoses, improving school supports, or increasing subsequent referral and access to appropriate therapies, interventions, or evaluations. Examples of these impacts are provided in Table 2. One study reported evidence that children enrolled on their neurodevelopmental follow‐up programme/care pathway achieved better receptive language scores than a similar historical control group; however, no differences were found for motor or behavioural scales.^51^ Similarly, when evaluated against a matched CHD comparison group, children completing neurodevelopmental follow‐up did not have greater service access of clinical therapies, despite receiving significantly more supports in the school setting.^35^ No other studies reported on the impact of their care pathway on developmental outcomes.

**TABLE 2:** Examples of reported outcomes of neurodevelopmental follow‐up programmes and care pathways for improving access to additional services and supports.

Study	Reported outcome
Chorna et al.^34^	Neurodevelopmental programme attendance generated first time referrals to early intervention services for 27% of children and to new additional services for 14% of children
Fourdain et al.^51^	Study demonstrated increased rates of referral to intervention services for children undergoing neurodevelopmental follow‐up (90%) compared with those receiving standard health care (37%)
Alam et al.^35^	Children started new speech therapy (50%), occupational therapy (56%), or physical therapy (50%) as a result of attending the neurodevelopmental follow‐up programme; 31% of children developed new school plans and 86% of struggling students improved grades
Tan et al.^47^	Children received a new autism diagnosis (*n* = 2) or referral to autism specialist (*n* = 9) as a result of neuropsychological evaluation in the follow‐up programme
Ruehl et al.^33^	The follow‐up programme supported 97% of children who required new or expanded education plans to achieve these. For those in the programme, 96% of children improved in at least one area of school performance

## DISCUSSION

In this scoping review we identified a range of structured programmes and care pathways for neurodevelopmental follow‐up of children with CHD. Although care pathways varied between studies, common elements reflected current guidance^2^, ^11^, ^57^ and included enrolment of children at high‐risk of neurodevelopmental delay into centralized clinics based in children's hospitals; referral or enrolment before hospital discharge; periodic follow‐up at fixed ages; standardized developmental assessment; and involvement of multidisciplinary teams. These care pathways and programmes generally had good rates of adoption by families and participation seemed to increase access to further evaluation and early intervention services. However, knowledge about whether these types of pathway optimize neurodevelopmental outcomes long‐term is still lacking. Notably, surveys of international practice demonstrated that structured neurodevelopmental follow‐up pathways and programmes for children with CHD were not routinely or widely implemented outside the USA. In the UK, Europe, South Africa, and Canada structured programmes did not exist in many locations and/or other care models were used.^14^, ^15^, ^16^, ^43^ Consequently, supporting the expansion of structured neurodevelopmental care pathways into these regions where services of this nature are not commonplace or exploring the effectiveness of delivering care in alternative ways remain important priorities.

Our review and evidence mapping also highlighted key barriers and enablers for delivering neurodevelopmental follow‐up. These provide a useful starting point for future research and can be used to develop targeted implementation strategies to increase adoption, scalability, and sustainability of these programmes. For example, the continuing lack of knowledge and awareness about neurodevelopmental follow‐up programmes and the AHA guidelines is a key barrier that should be addressed by implementation strategies that train and educate stakeholders or develop stakeholder interrelationships.^58^ Additionally, practice surveys showed that not all primary care providers or paediatric cardiologists had access to neurodevelopmental follow‐up programmes for their patients. Better collaboration with primary care, and other existing services (including neonatal follow‐up or community‐based developmental providers), should be considered a key priority in addressing this issue. Increased collaboration will also assist in leveraging resources in fiscally challenging health‐care environments. Finally, costs featured repeatedly as key implementation barriers. However, there is a paucity of research that has examined the cost of delivering neurodevelopmental follow‐up care, or the cost‐effectiveness of implementing such care pathways and subsequent interventions for children diagnosed with neurodevelopmental disorders.^10^, ^59^ Future research that quantifies the comparative benefits (or otherwise) of investing in both structured neurodevelopmental follow‐up care pathways for high‐risk children with CHD and broader surveillance approaches in this population remains a priority.

There may be additional opportunities to learn from more established systems of neurodevelopmental follow‐up for high‐risk populations, such as those for infants born preterm. However, like our findings, the heterogeneity in the structure and processes of these programmes and pathways makes this difficult, with little to no evidence of the extent of implementation of guidelines in practice and their consequent effectiveness.^60^, ^61^ Furthermore, funding has been identified as a major barrier to these programmes' sustainability and scalability. Concerningly, as is the case with CHD, most of the literature on neurodevelopmental follow‐up of preterm birth originates from a few selected high‐income countries, with almost no evidence from countries and regions with some of the highest rates of preterm birth.^62^ There is, however, emerging evidence that quality improvement learning collaboratives can aid in reducing variability in care practices and provide opportunities for improving compliance with follow‐up.^63^, ^64^ Finally, new models of delivery of neonatal follow‐up care such as the nurse‐led model, community health‐care provider model, or the hybrid virtual model look promising and may have application in children with CHD.^65^, ^66^, ^67^

Several publications provide recommendations for delivering neurodevelopmental follow‐up to children with CHD. The impact of the AHA's 2012 Scientific Statement for evaluation and management of children with CHD can be observed in this review. For example, two‐thirds of the clinical sites only established care pathways or programmes after publication of this guidance, and most seem to follow its key recommendations. This includes use of a skilled multidisciplinary team; providing intervention recommendations; medical, developmental, and psychosocial review complemented by age‐specific standardized testing; and referral of children to early intervention services. Additionally, the most used assessment instruments align with the AHA recommendations. These included the Bayley Scales of Infant and Toddler Development, Third Edition (considered the most comprehensive developmental assessment tool for determining delays in the early years), Adaptive Behaviour Assessment System, Child Behaviour Checklist, and Behaviour Assessment System for Children.

Two more recent publications have extended this initial guidance, outlining consensus‐based Neurodevelopmental Assessment Batteries for children with complex CHD aged 0 to 5 years^11^ and of school age.^57^ The core versions of these batteries contain an abbreviated set of assessments considered the minimum required for adequate developmental evaluation. However, none of the studies included in our review reported using the full set of core instruments in their assessment process. In fact, several core instruments were not used by any programme or care pathway included in our review. The absence of these core assessments in included studies may simply represent a lag in translation of guidelines to practice given most data collection for included studies occurred before their publication. Nevertheless, adoption of these new core instrument sets across clinical sites and care pathways should be encouraged for performing large‐scale collaborative research, evaluating outcomes, and conducting benchmarking and quality improvement exercises.

However, widescale adoption of extensive testing batteries should also be tempered with consideration of their impacts on health services, providers, and families. Limited resources, staff shortages, and competing time demands may present implementation challenges in clinical settings. Most of these tests also necessitate attendance face‐to‐face at specialized clinics, particularly as providers have raised concerns about the practicality and validity of some instruments for teleassessment.^46^ Consequently, it is crucial that children who undergo more frequent or extensive developmental assessment experience positive benefits that outweigh any additional burden such as time off school or work, financial costs, or stress.^68^ Unfortunately, research into the long‐term neurodevelopmental trajectories of children with CHD who have access to structured follow‐up is lacking. However, this review provides evidence that these programmes appropriately support parents, and route children and families to needed intervention services. Consequently, it seems reasonable to suggest that such an approach would confer similar benefits as seen in preterm and other populations by promoting parental involvement^69^ and accessing early interventions^70^ and occupational therapy.^71^ However, given the lack of evidence comparing the effectiveness of different follow‐up practices, a balance needs to be maintained between performing extensive assessment and minimizing burden on families.

Alternative approaches to centre‐based structured neurodevelopmental follow‐up of all at‐risk children with CHD are emerging. The most common approach proposes screening at‐risk children with simpler tools in the first instance, supported by referral for an extended assessment when problems are identified.^72^, ^73^, ^74^ For example, the Brief Developmental Assessment has been developed and piloted for use by non‐specialists as an early recognition tool of developmental delay.^75^ It has demonstrated acceptability for clinicians and parents and has potential for application in teleassessment.^76^ The adoption of telephone or locally‐based screening by parents and primary care providers (before triage to formal evaluation) was also observed in included studies from the USA,^34^ France,^28^ and Australia.^53^ Finally, the use of self‐ or parent‐report questionnaires (such as Ages and Stages) as a follow‐up approach has also been endorsed by young people with CHD.^68^ Consequently, research that quantifies and compares the benefits and burdens of these various care pathways is needed to move away from a one‐size‐fits‐all approach to contextually relevant and family relevant service delivery.

Another notable gap we observed in neurodevelopmental follow‐up was the assessment of mental health of primary caregivers. Evidence suggests that parental mental health may play a key role in neurodevelopmental outcomes in children with CHD.^56^ With the reported increased risk for poorer mental health and well‐being for parents of children with CHD,^77^ there is sound rationale for parental screening as an important component of neurodevelopmental follow‐up care.^11^, ^56^ Availability of psychological care concurrent with their child's medical review may also reduce barriers to service access for parents, through minimized appointment numbers and familiarity with care providers.^78^ Yet, only one study^53^ identified in this review explicitly reported routine screening of parental mental health as a neurodevelopmental care pathway component. Additionally, only three programmes (38%) in the survey of Canadian follow‐up practice^15^ reported similar screening practices. Increasing parental mental health screening in neurodevelopmental follow‐up and cardiac care more broadly^77^, ^79^ should therefore continue to be a priority for implementation.

It is important to note that the content of research publications, which are the focus of this review, will not necessarily represent all neurodevelopmental follow‐up programmes and pathways available in routine practice. For example, the included publications capture individual care pathways or programmes at only 10 of the 47 institutional members of the Cardiac Neurodevelopmental Outcome Collaborative, the key multicentre and multinational group operating in this field. Additionally, the practice surveys/overviews highlight key programmes in Europe,^16^, ^30^ Canada,^15^ and South Africa,^14^ yet only five studies from these regions reported care pathways eligible for inclusion in this review. Consequently, there probably remains opportunity to enhance care internationally through further research publication and formal and informal clinical networks and communities of practice.

Further research across geographical and organizational contexts is required to ensure advancements in neurodevelopmental follow‐up are both evidence‐based and locally relevant. Much of the research evidence so far comes from the USA, with some of the key challenges highlighted likely to be specific to that health system (e.g. patients' insurance coverage). Additionally, we did not identify any studies in low‐ or middle‐income countries, except for South Africa. Knowledge about how programmes and care pathways are implemented in health‐care systems with different private–public service mixes, out‐of‐pocket costs, integration of services, and funding models is still unknown. Moreover, given that travel to centralized clinics to attend follow‐up was a key implementation barrier, regions with a large geographical dispersion of families from the surgical centre may require alternative models of delivery. However, at the current time, telehealth‐only models of care do not seem appropriate to fill this gap. Interestingly, two decentralized care pathways were reported by programmes in Canada and Australia, both countries with comparable distributions of urban and regional areas, similar national travel times to cities, and significantly lower population densities than the USA.^80^, ^81^ Understanding how evidence‐based care pathways can be adapted in these settings (and others) is important given that application of AHA guidelines in international contexts was identified as a key challenge in this review.

Surveys of neurodevelopmental follow‐up in Canada, Europe, and the UK highlighted the importance of having evidence‐based guidance to support standardization across geographical contexts, but which was also relevant to the local setting.^15^, ^16^, ^43^ Guidelines produced in one setting may not always be appropriate for use in another, even with the same underpinning evidence. Consideration needs to be given to how specific patient preferences, legislation, resources, cultural contexts, population characteristics, and organization of health services differ in the new setting.^82^ Given the relatively small and concentrated evidence base in the field, adaptation of the existing guidelines (rather than development of new country‐specific guidelines) may be preferable to reduce duplication of effort in guideline development. Locally tailored guidelines may aid in adherence to and acceptance of the recommendations.^83^ If guideline adaption includes engagement with neurodevelopmental follow‐up care providers, it may also increase local buy‐in and allow targeted implementation strategies. This is important given the high levels of practice variability and low rates of guideline awareness highlighted in this review.

Our review highlighted the current lack of comparative or interventional studies examining the impact of neurodevelopmental follow‐up on outcomes for children with CHD. The implementation studies included in this review were mostly single‐centre, retrospective, and observational in design. This is consistent with the lack of empirical data about effectiveness of these care pathways recently identified in the Cardiac Neurodevelopmental Outcome Collaborative research agenda.^10^ Consequently, we concur with this agenda about the need for more comparative research in this field, particularly interventional trials of different follow‐up approaches measuring long‐term outcomes of children and families. Achieving this through single‐centre approaches will be challenging, and highlights the critical need for collaborative initiatives, including those that enable comparison between varying programme and care pathway models. Further to this, collaborations inclusive of family and advocacy group partnership are needed to ensure both what is captured and how outcomes are documented are meaningful.^12^, ^84^

Formal qualitative approaches to understand both provider and family experiences of neurodevelopmental follow‐up care were also lacking from the included studies. This is important as qualitative approaches can allow exploration of the acceptability of care from multiple perspectives, and how a particular programme or care pathway may have contributed to improved outcomes and why.^85^ This information can then be used to refine programmes, encourage greater uptake, or improve effectiveness and sustainability. Additionally, there is an increasing interest in incorporating patient experience into health‐care decision‐making, including in neurodevelopmental care of children with CHD,^84^, ^86^ given its association with improved health outcomes, decreased health‐care use, and improved treatment adherence.^87^, ^88^ Although data about patient reported experience were limited in studies included our review, the high rates of programme uptake and parental satisfaction that were reported highlight that families see neurodevelopmental follow‐up as an important issue for their children.

Process evaluations offer a feasible approach of using qualitative methodologies about stakeholder experience, yet these too were absent from the included studies. Process evaluation should be an important component of implementation research in this field as it provides information about why and how a programme works in a real‐world context, and what factors may have impacted outcomes.^89^ It can also be used to explore adaptations that may be required to make programmes more culturally appropriate for patients. Given the variability in approaches seen in the literature, context‐specific barriers identified, and diversity of settings and patients, future evaluations of neurodevelopmental follow‐up should consider an embedded process evaluation. Moreover, such evaluations should incorporate mixed‐method qualitative and quantitative approaches with a focus on patients' experiences.

Economic evaluation can also provide important evidence for health‐care decision‐makers about the potential effects and costs of an intervention or model of care. This is important for achieving favourable resource allocation in the current climate of balancing finite budgets with increasing demand for health services.^90^ Notably, however, none of the included studies in this review provided outcomes or costs that could be used for economic evaluation of neurodevelopmental follow‐up care for children with CHD. In part this may be explained by the difficulties inherent in measuring outcomes in this population, when the impact is seen across physical, social, psychological, and cognitive domains. Importantly, the outcome measures chosen should be clinically meaningful to children and families.^10^ Measurement of health‐related quality of life may best reflect the changes in well‐being of children with CHD that may occur through neurodevelopmental care. However, ascertaining this either from general health profile measurement tools or from ones that are disease‐specific has proved problematic so far.^59^ Additionally, given that costs (to families and the health system) featured as a key implementation barrier in individual programmes, pathways, and surveys in this review, more work needs to be done to accurately quantify the cost of providing neurodevelopmental follow‐up care. Measuring such costs will involve the systematic identification and measurement of processes and resources used throughout the care pathway in addition to the costs borne by families as their child receives care. Consequently, further research to progress cost‐effectiveness analyses of programmes with measurement of long‐term outcomes is encouraged.

Consistent with publications of other non‐pharmaceutical interventions,^91^, ^92^ complete descriptions of neurodevelopmental programmes and care pathways were mostly missing from the included studies. It was not always clear what a follow‐up clinic appointment involved, what assessments were performed, and what other supports were provided (e.g. education or feedback). These limitations in reporting make it challenging to accurately define components of a successful programme and understand their relationship to outcomes. Additionally, given neurodevelopmental follow‐up can be conceptualized as a complex intervention, clear and complete reporting of processes is important for informed decision‐making about how such a programme or care pathway could be implemented in a new clinical setting. Finally, describing the programme components in detail allows a nuanced assessment of the costs involved with service delivery. Fortunately, widely accepted guidance is available to assist authors in transparently and consistently describing components of their neurodevelopmental programmes/care pathways.^93^ Although the use of the Template for Intervention Description and Replication checklist has so far largely been limited to reporting trial interventions, it has applicability across diverse research designs, including programme evaluation.^94^ We encourage authors of neurodevelopmental follow‐up research to use this guidance in describing their programmes and care pathways.

Our review has notable strengths. To our knowledge, this is the first comprehensive review of the published literature about neurodevelopmental follow‐up care for children with CHD. It builds on earlier work characterizing these programmes^12^ and highlights the continuing challenges still relevant to the field. In addition, it takes a broader perspective not only describing the elements of different care pathways but also considering clinical and health service outcomes, implementation determinants, and multiple surveys of current international practice. The review has been enhanced by diverse perspectives from a multidisciplinary team with both clinical and methodological expertise. However, it is not without limitations. First, although our search methodology was extensive, we may have missed including follow‐up care pathways or programmes that have been described outside published peer‐reviewed sources (e.g. in theses, institutional reports, or websites). Second, considering the heterogeneity of the included studies, their small sample sizes, and observational designs, outcome data must be interpreted with caution. Finally, there is no evidence comparing different care pathways, limiting our ability to draw conclusions about an optimal care pathway.

## CONCLUSION

Published descriptions of neurodevelopmental follow‐up programmes and care pathways for children with CHD, including their implementation and impact, are limited and constrained to a few geographical regions. Defining components of a successful care pathway and understanding how they may be adapted to different contexts remains a priority. Consequently, neurodevelopmental follow‐up providers should be encouraged to evaluate and report on their programmes. Future research using larger collaborative studies is likely to be beneficial for examining the comparative outcomes of different care pathways. Particular attention should be paid to describing the experience of families, implementation processes, and cost‐effectiveness to provide critical insights into the gaps identified in this review.

## FUNDING INFORMATION

Medical Research Future Fund Congenital Heart Disease Grant (ARGCHDG0035) 2020–2024. This funder provided partial salary for BRA.

## Supporting information


**Appendix S1:** Scoping review protocol.

**Appendix S2:** Search strings for OVID Medline, Embase, CINAHL, and Scopus.

**Figure S1:** PRISMA flow diagram of search and study selection process.

**Figure S2:** Visual representation of the relationship between publications, clinical centres/sites, and care pathways.

**Table S1:** Keyword and MeSH terms for initial search strategy.

**Table S2:** Characteristics of included studies reporting on implementation and/or evaluation of individual neurodevelopmental follow‐up programs and pathways.

**Table S3:** Characteristics of included studies reporting on surveys or overviews of neurodevelopmental follow‐up practices.

**Table S4:** Detailed descriptions of programs and care pathways for neurodevelopmental follow‐up of children with CHD reported in included studies.

**Table S5:** Detailed descriptions of international practices and overviews of neurodevelopmental follow‐up of children with CHD reported in included surveys.

## Data Availability

The original studies/data that support the findings of this study are available in the supplementary material of this article. Data extracted from these studies are available from the corresponding author upon reasonable request.
