# Supplementary material for: Implementing neurodevelopmental follow‐up care for children with congenital heart disease: A scoping review with evidence mapping
Source: Dev Med Child Neurol. 2023 Jul 8;66(2):161–75. doi: 10.1111/dmcn.15698 (PMC10953404; doi:10.1111/dmcn.15698)
Supplement: Supplementary file 1 — Appendix S1: Scoping review protocol. [file DMCN-66-161-s004.pdf]

Characteristics, Barriers, and Enablers of Programs Supporting Neurodevelopmental Follow-up of Children with Congenital Heart Disease: A Scoping Review Protocol

Introduction

Congenital heart disease (CHD) includes a series of structural cardiac abnormalities that arise before birth due to abnormal fetal cardiac development. Advances in both the diagnosis and treatment of children with CHD mean that more than 90% of children born with complex congenital heart disease will now survive into adulthood[1]. Consequently, with the growing number of CHD survivors there has been an increased focus on assessing and understanding long-term morbidity in this population. This morbidity is commonly manifested as a range of cognitive, learning, motor, and psychosocial impairments characterised as developmental delays or disabilities[2]. Up to 50% of children requiring intervention for CHD will experience such adverse developmental outcomes[3]. While some of these impairments may be mild or remediated over time, for many children developmental challenges can be cumulative and persist long-term[3, 4]. Adverse impacts can be observed on education, employment, independence and quality of life[3]. Consequently, ongoing surveillance, screening, and early identification of developmental delays in children with CHD is an important approach to optimise neurodevelopmental outcomes for this population.

A three-layered model of increasing intensity has been proposed by the American Heart Association (AHA) to provide neurodevelopmental follow-up to children with CHD[1]. In the first instance, routine developmental *surveillance* or monitoring should be provided to all children with CHD by their primary care provider. A more comprehensive follow-up approach of *screening* with standardised tests or scales should be adopted for children with CHD who are identified as at a greater risk of adverse developmental outcomes (due to their diagnosis or other concerns). Finally, specialised *evaluation* can be performed by trained professionals to provide a developmental or behavioural diagnosis and enable access to intervention services. Since the publication of these AHA recommendations, neurodevelopmental follow-up programs have emerged as way of providing both structured screening and evaluation to this population[5, 6].

Neurodevelopmental follow-up programs aim to provide comprehensive medical and developmental care to children with CHD by translating research evidence into clinical practice.[7] Programs have traditionally been run out of hospital centres providing surgical interventions for CHD. They usually include multidisciplinary teams of providers, with elements such as care coordination, developmental assessment, provision of education and resources, and referral to therapies. Despite the increasing role these programs play in providing long-term neurodevelopmental follow-up for children with CHD, limited research considers the design and implementation of such programs[6]. Understanding the different program approaches and models of care that can be effectively adopted is critical to guide implementation, scale-up and evaluation of their impacts in practice [6]. Yet, to date, these different approaches have not been well characterised or compared in published literature. One paper published in 2014 provides a comprehensive overview of key components, implementation processes and challenges of existing neurodevelopmental follow-up programs at that time[8]. However, our preliminary searches (MEDLINE, Google Scholar: conducted in August 2021) have not identified any systematic or scoping reviews of this literature since. Moreover, surveys of current practice have highlighted the considerable variability that exists in neurodevelopmental follow-up care both within and across countries[9-11].

The objectives of the scoping review are to systematically identify literature describing components of neurodevelopmental follow-up programs for children with congenital heart disease, categorise the described models of care and outcomes, and highlight contextual barriers and enablers impacting implementation.

Methods

A scoping review was considered to be the most suitable approach which aligned with the aim of producing a broad overview of the field of neurodevelopmental follow-up for children with CHD.

Review team

The review is being conducted by a team of multidisciplinary academics and expert clinicians in the field of pediatrics, cardiology, and health services research.

Scoping review framework

This scoping review will follow the methodology proposed by Arksey and O'Malley[12] and subsequent amendments by Levac, Colquhoun and O'Brien[13]. This includes five stages:

(1) identifying the research question,

(2) identifying relevant studies,

(3) study selection,

(4) charting the data, and

(5) collating, summarising and reporting results.

As this is a scoping review it will be designed to identify the range of the programs available, represented as a mapping of the identified data, rather than using meta-analysis or meta-synthesis. Consequently, assessment of methodological quality will not be performed.

The Preferred Reporting Items for Systematic Reviews and Meta-Analysis extension for scoping reviews (PRISMA-ScR)[14] and JBI Manual for Evidence Synthesis[15] will be used to guide the reporting of the scoping review.

Stage 1: Identifying the research question (completed)

The research question for the scoping review forms part of the evidence synthesis phase of a broader research project aiming to understand current evidence and local practices and create a taxonomy of care for neurodevelopmental follow-up of children with CHD. In this review the research question has been developed by the review team based on this larger project and identified gaps in the literature. Consequently, this review asks: *What programs are reported in international literature for the neurodevelopmental follow-up of children with congenital heart disease?*

Stage 2: Identifying the relevant studies (ongoing)

Studies for inclusion will be identified through electronic bibliographic database searching supplemented by forwards citation tracking and consultation with key clinical experts. Based on guidance from the JBI manual, broad search terms were used in Google Scholar and MEDLINE to identify relevant publications. The titles, abstracts and keywords of these publications were used to develop an initial search which was then piloted in Search Refinery to assess relevance and coverage of keywords and MeSH terms and iteratively refined (see Table 1 to Table 3).

Inclusion criteria are based on the 'Population-Concept-Context' (PCC) of the review.

Population

Infants, children, or youth (0-18 years old) who have diagnosed congenital heart disease and have undergone surgical intervention. *Note: refined to infants, children, or youth (0-18 years old) who have diagnosed congenital heart disease during title and abstract screening process to broaden scope to reflect more common eligibility criteria for follow-up programs in practice, and AHA guidelines for "at-risk" children.*

Concept/ phenomena of interest

Description of a model of care, process, or program for neurodevelopmental or developmental follow-up, screening, surveillance, or assessment of the population of interest and/or their families

Context

Decided was not applicable during initial searching strategy design process

Table 1. Draft initial search strategy based on P-C-C

**Population**<br>*Important characteristics of participants, including age and other qualifying criteria*		**Concept/phenomena of interest**<br>*The core concept examined by the scoping review should be clearly articulated to guide the scope and breadth of the inquiry, such as the "interventions" and/or "phenomena of interest" and/or "outcomes*	
**Age: Infants, children, youth**	**Condition: congenital heart disease with surgical intervention**	**Models of care for neurodevelopmental assessment, screening, follow-up**	
"Child"[Mesh]<br>"Infant"[Mesh]<br>"Adolescent"[Mesh]<br>Pediatrics[Mesh]<br><br>child*[tiab]<br>infant*[tiab]<br>adolescen*[tiab]<br>teenage*[tiab]<br>p$ediatr*[tiab]<br>teen*[tiab]<br>youth[tiab]<br>kids[tiab]<br>newborn[tiab]<br>famil*[tiab]<br>neonat*[tiab]<br>prematur*[tiab]<br>$school[tiab]<br>toddler[tiab]	"Heart Defects, Congenital"[Mesh]<br>"fetal heart"[Mesh]<br><br>congenital[tiab] AND heart disease*[tiab]<br>congenital*[tiab] AND heart defect*[tiab]<br><br>heart abnormalit*[tiab]<br>heart malformation*[tiab]<br>heart disease*[tiab]<br>cardiac abnormalit*[tiab]<br>cardiac malformation*[tiab]<br>cardiac diseas*[tiab]<br>cardiac defect*[tiab]<br>CHD [tiab]<br><br>Surgery AND Heart OR cardiac [tiab]	"Mass screening"[Mesh]<br>"Epidemiologic Studies"[Mesh]<br>Referral and Consultation[Mesh]<br><br>Early intervention[tiab]<br><br>"delivery of health care"[Mesh]<br>"quality of health care"[Mesh] *<br>"Outcome Assessment, Health Care"[Mesh]	"Child Development"[Mesh]<br>"Cognition Disorders"[Mesh]<br>"Neurodevelopmental Disorders"[Mesh]<br>"Neuropsychological Tests"[Mesh]<br>"Intelligence Tests"[Mesh]<br><br>neurodevelopment*[tiab] AND evaluat* OR outcome* or risk or assess* or test* OR follow-up or delay* OR disabilit* OR disorder* OR impair* OR limit* OR problem* OR function* OR change* OR prognosis OR screen* OR examin* OR refer* OR tool* OR surveillance OR therap* OR intervent* OR program* OR model* OR care<br>866 hits with just neurodevelopment or 859 narrowed<br><br>development* AND evaluat* OR outcome* or risk or assess* or test* OR follow-up or delay* OR disabilit* OR disorder* OR impair* OR limit* OR problem* OR function* OR change* OR prognosis OR screen* OR examin* OR refer* OR tool* OR surveillance OR therap* OR intervent* OR program* OR model* OR care<br>12968<br><br>neuropsychological<br>neurocognit*[tiab]<br>neurobehavi*[tiab]<br>neuromotor*[tiab]<br>neurologic*[tiab]

Table 2. Refined strategy with low-value/high-noise terms ~~removed~~, and key terms highlighted in green and terms with few hits highlighted in red (using search refiner)

**Population**<br>*Important characteristics of participants, including age and other qualifying criteria*		**Concept/phenomena of interest**<br>*The core concept examined by the scoping review should be clearly articulated to guide the scope and breadth of the inquiry, such as the "interventions" and/or "phenomena of interest" and/or "outcomes*	
**Age: Infants, children, youth**	**Condition: congenital heart disease with surgical intervention**	**Models of care for neurodevelopmental assessment, screening, follow-up**	
"Child"[Mesh]<br>"Infant"[Mesh]<br>"Adolescent"[Mesh]<br>Pediatrics[Mesh]<br><br>child*[tiab]<br>infant*[tiab]<br>adolescen*[tiab]<br>teenage*[tiab]<br>p$ediatr*[tiab]<br>~~teen*[tiab]~~<br>~~youth[tiab]~~<br>~~kids[tiab]~~<br>~~newborn[tiab]~~<br>famil*[tiab]<br>neonat*[tiab]<br>prematur*[tiab]<br>$school[tiab]<br>toddler[tiab]<br>~~Prenatal~~<br>~~Postnatal~~	"Heart Defects, Congenital"[Mesh]<br>~~"fetal heart"[Mesh]~~<br><br>congenital[tiab] AND heart disease*[tiab]<br>~~congenital*[tiab] AND heart defect*[tiab]~~<br><br>~~heart abnormalit*[tiab]~~<br>~~heart malformation*[tiab]~~<br>~~heart defect*[tiab]~~<br><br>heart disease*[tiab]<br>~~cardiac abnormalit*[tiab]~~<br>~~cardiac malformation*[tiab]~~<br>cardiac diseas*[tiab]<br>~~cardiac defect*[tiab]~~<br>CHD [tiab]<br>Heart OR ~~cardiac~~ AND surgery	"Neuropsychological Tests"[Mesh]<br>"Mass screening"[Mesh]<br>"Epidemiologic Studies"[Mesh]<br>Referral and Consultation[Mesh]<br><br>Early intervention[tiab]<br><br>Developmental Disabilities if not exp nd<br><br>~~cog dis~~<br>~~lang dis~~<br>~~psychomotor performance"[MeSH~~<br>~~pysc disorders~~<br>neuropsychological<br>neuro cognit*[tiab]<br>~~neurobehavi*[tiab]~~<br>~~neuromotor*[tiab]~~<br>~~neurologic*[tiab]~~	"delivery of health care"[Mesh]<br>"quality of health care"[Mesh] *<br>"Outcome Assessment, Health Care"[Mesh]<br>"Child Development"[Mesh]<br>"Cognition Disorders"[Mesh]<br>"Neurodevelopmental Disorders"[Mesh]<br><br>neurodevelopment*[tiab] AND evaluat* OR outcome* or risk or assess* or test* OR follow-up or delay* OR disabilit* OR disorder* OR impair* OR limit* OR problem* OR function* OR prognosis OR screen* OR examin* OR refer* OR tool* OR surveillance OR therap* OR intervent* OR program* OR model* OR care<br>866 hits with just neurodevelopment or 859 narrowed<br><br>~~development* AND evaluat* OR outcome* or risk or assess* or test* OR follow-up or delay* OR disabilit* OR disorder* OR impair* OR limit* OR problem* OR function* OR change* OR prognosis OR screen* OR examin* OR refer* OR tool* OR surveillance OR therap* OR intervent* OR program* OR model* OR care~~<br>~~12968~~<br><br>neuropsychological<br>neurocognit*[tiab]<br>~~neurobehavi*[tiab]~~<br>~~neuromotor*[tiab]~~<br>~~neurologic*[tiab]~~

Table 3. Further refinement of strategy with search refiner to find all pilot studies and reduce irrelevant search terms

**Population**<br>***Important characteristics of participants, including age and other qualifying criteria***		**Concept/phenomena of interest**<br>***The core concept examined by the scoping review should be clearly articulated to guide the scope and breadth of the inquiry, such as the "interventions" and/or "phenomena of interest" and/or "outcomes***
**Age: Infants, children, youth**	**Condition: congenital heart disease with surgical intervention**	**Models of care for neurodevelopmental assessment, screening, follow-up**
"Child"[Mesh/exp]<br>"Infant"[Mesh/exp]<br>"Adolescent"[Mesh]<br><br>child*[tw]<br>infant*[tw]<br>famil*[tw]<br>$school[tw]<br>P?ediatric*[tw]<br>Premature*[tw]	"Heart Defects, Congenital"[Mesh]<br><br>Congenital heart disease*[tw]<br>CHD [tw]<br>Congenital heart [tw]<br>Heart ADJ1 surgery [tw]	"Neurodevelopmental Disorders"[Mesh/exp]<br>"Child Development"[Mesh]<br>Referral and Consultation[Mesh]<br>"delivery of health care"[Mesh]<br>Cognition disorders[Mesh]<br>Language disorders[Mesh]<br>Psychomotor disorders[Mesh]<br><br>Early intervention[tw]<br>Neurodevelopment* [tw]<br><br>neurodevelopment* AND outcome* or assess* or test* OR follow-up or delay* OR impair* OR screen* OR examin* OR refer* OR intervent* OR disorder* OR program* OR care [tw]

From this a comprehensive search strategy for MEDLINE was developed and refined in order to identify all relevant literature in databases:

(exp Child/ OR exp Infant/ OR Adolescent/ OR child.tw. OR infant*.tw. OR famil*.tw. OR ?school.tw. OR p?ediatr*.tw. OR prematur*.tw.) AND (Heart Defects, Congenital/ OR "congenital heart disease*".tw. OR CHD.tw. OR congenital heart.tw. OR (heart ADJ1 surgery).tw.) AND (exp Neurodevelopmental Disorders/ OR Child Development/ OR "Referral and Consultation"/ OR "Delivery of Health Care"/ OR Cognition Disorders/ OR Language Disorders/ OR Psychomotor Disorders/ OR "early intervention".tw. OR neurodevelopment*.tw.)*

It will be adapted to the requirements of each database searched. These include MEDLINE, Scopus, EMBASE and CINAHL. The final list of included studies will be subject to forward citation tracking to identify any subsequently published studies for screening and inclusion. Finally, the expert clinicians on the review team will be asked to identify any additional publications for inclusion in the review.

Stage 3: Study selection (not yet started)

The PCC framework and additional eligibility criteria related to the research question will guide title and abstract screening.

Inclusion criteria

Meets the PCC criteria. We will include all types of evidence such as primary research, systematic reviews, narrative reviews, guidelines, policy documents, websites, conference papers and theses. These may be of qualitative, quantitative or mixed methods designs. Note: *during the title and abstract screening process decided to exclude non-peer reviewed sources such as websites and theses to capture higher quality evidence. At full-text stage decided to exclude policy/guidelines as we were more concerned with understanding what was happening in current practice rather than what the recommendations were.* Publications meeting all these inclusion criteria **should not be** excluded based on language, publication date, or geographical region.

Exclusion criteria

1. Population of focus is children with congenital heart disease without surgical intervention *(removed this exclusion criteria during title and abstract screening)*

2. Population of focus is children with acquired heart disease
3. Population of focus is adults with congenital heart disease
4. Settings providing developmental support outside the health care sector, including education and disability providers
5. Publication is a duplicate reference/duplicate abstract

All identified records will be uploaded into Endnote software, and any duplicates identified and removed. This reference library will then be exported and uploaded to the EPPI-reviewer platform for title, abstract and full-text screening.

At least two reviewers will independently perform screening of each record at both the title/abstract and full-text stages. Discussion and resolution of differing opinions will occur in regular review team meetings.

Stage 4: Charting the data (not yet started)

A data charting spreadsheet created in Excel will be used to electronically capture relevant information from each included study. This spreadsheet has been created at the outset of the review and will be iteratively refined through the review to ensure it captures relevant information from included studies. It will contain the following information: publication characteristics (e.g., author, year, date), neurodevelopmental pathway/program components (e.g., referral, eligibility, scheduling, location, providers, partnerships, service structure, therapy/intervention, caregiver assessment, registry, length of program), and outcomes reported (e.g., costs, adoption, acceptability, barriers/enablers, safety, quality of life, access, clinical improvements).

At least two reviewers will independently extract data from each included study. Discussion and resolution of any issues or differing opinions will occur in regular review team meetings.

Stage 5: Collating, summarising and reporting of results

Results will be presented as a narrative summary with supporting tables. Both qualitative and quantitative data will be reported. A matrix/evidence map will be developed to categorise and visualise information about characteristics of individual follow-up programs. Qualitative data will be reported thematically, mostly related to barriers and enablers of program implementation. PRISMA-ScR guidance will be followed for reporting the review.

Study status

The database search strategy has been developed and piloted. Database searches are about to commence.

Dissemination and ethics

The findings of the review will be presented at conferences and published in a peer-reviewed journal. This scoping review and evidence map will provide clinicians and researchers with an overview of the currently published international literature describing characteristics of neurodevelopmental programs for children with CHD. This will additionally inform research on the design, implementation, and effectiveness of such programs in the future.

As all data will be obtained from publicly available documents, ethical approval is not required for the review.

References

1. Stout KK, Daniels CJ, Aboulhosn JA, et al. 2018 AHA/ACC guideline for the management of adults with congenital heart disease: a report of the American College of Cardiology/American Heart Association Task Force on Clinical Practice Guidelines. *Journal of the American College of Cardiology* 2019; 73: e81-e192.
2. Marino BS, Lipkin PH, Newburger JW, et al. Neurodevelopmental outcomes in children with congenital heart disease: evaluation and management: a scientific statement from the American Heart Association. *Circulation* 2012; 126: 1143-1172.
3. Verrall CE, Blue GM, Loughran-Fowlds A, et al. 'Big issues' in neurodevelopment for children and adults with congenital heart disease. *Open Heart* 2019; 6: e000998.
4. Marelli A, Miller SP, Marino BS, et al. Brain in Congenital Heart Disease Across the Lifespan: The Cumulative Burden of Injury. *Circulation* 2016; 133: 1951-1962. 2016/05/18. DOI: 10.1161/circulationaha.115.019881.
5. Knutson S, Kelleman MS and Kochilas L. Implementation of developmental screening guidelines for children with congenital heart disease. *The Journal of Pediatrics* 2016; 176: 135-141. e132.
6. Cassidy AR, Butler SC, Briend J, et al. Neurodevelopmental and psychosocial interventions for individuals with congenital heart disease: A research agenda and recommendations from the Cardiac Neurodevelopmental Outcome Collaborative. *Cardiology in the Young* 2021; 31: 888.
7. Ware J, Butcher JL, Latal B, et al. Neurodevelopmental evaluation strategies for children with congenital heart disease aged birth through 5 years: recommendations from the cardiac neurodevelopmental outcome collaborative. *Cardiology in the Young* 2020; 30: 1609-1622.
8. Brosig C, Butcher J, Butler S, et al. Monitoring developmental risk and promoting success for children with congenital heart disease: Recommendations for cardiac neurodevelopmental follow-up programs. *Clinical Practice in Pediatric Psychology* 2014; 2: 153.
9. Miller TA, Sadhwani A, Sanz J, et al. Variations in practice in cardiac neurodevelopmental follow-up programs. *Cardiology in the Young* 2020; 30: 1603-1608.
10. Smith R, Le Roux H, Nel H, et al. Neurodevelopmental evaluation and referral practices in children with congenital heart disease in central South Africa. *SA Heart* 2019; 16: 324-332.
11. Di Maria MV, Brown DW, Cetta F, et al. Surveillance testing and preventive care after Fontan operation: a multi-institutional survey. *Pediatric cardiology* 2019; 40: 110-115.
12. Arksey H and O'Malley L. Scoping studies: towards a methodological framework. *International journal of social research methodology* 2005; 8: 19-32.
13. Levac D, Colquhoun H and O'Brien KK. Scoping studies: advancing the methodology. *Implementation science* 2010; 5: 1-9.
14. Tricco AC, Lillie E, Zarin W, et al. PRISMA extension for scoping reviews (PRISMA-ScR): checklist and explanation. *Annals of internal medicine* 2018; 169: 467-473.
15. JBI Manual for Evidence Synthesis. In: Aromataris E and Munn Z, (eds.). JBI, 2020.
