# Supplementary material for: Implementing neurodevelopmental follow‐up care for children with congenital heart disease: A scoping review with evidence mapping
Source: Dev Med Child Neurol. 2023 Jul 8;66(2):161–75. doi: 10.1111/dmcn.15698 (PMC10953404; doi:10.1111/dmcn.15698)
Supplement: Supplementary file 2 — Appendix S2: Search strings for OVID Medline, Embase, CINAHL, and Scopus. [file DMCN-66-161-s003.pdf]

Supporting Information S2. Search strings for OVID Medline, Embase, CINAHL and Scopus

OVID Medline search string (16 August 2021 and 17 March 2023)

((exp Child/ OR exp Infant/ OR Adolescent/ OR child*.tw. OR infant*.tw. OR famil*.tw. OR ?school.tw. OR p?ediatr*.tw. OR prematur*.tw.) AND (Heart Defects, Congenital/ OR “congenital heart disease*".tw. OR CHD.tw. OR congenital heart.tw. OR (heart ADJ1 surgery).tw.) AND (exp Neurodevelopmental Disorders/ OR Child Development/ OR "Referral and Consultation"/ OR “Delivery of Health Care”/ OR Cognition Disorders/ OR Language Disorders/ OR Psychomotor Disorders/ OR "early intervention”.tw. OR neurodevelopment*.tw.)) NOT (mice or mouse or rat OR rats or animal or gene or genes OR phenotype or cell Or mutation).tw.)

Embase search string (16 August 2021 and 17 March 2023)

(Child/exp OR Infant/exp OR Adolescent/de OR child*:ti,ab,kw OR infant*:ti,ab,kw OR famil*:ti,ab,kw OR school:ti,ab,kw OR p?ediatr*:ti,ab,kw OR prematur*:ti,ab,kw) AND ("congenital heart malformation"/exp OR "congenital heart disease*":ti,ab,kw OR CHD:ti,ab,kw OR "congenital heart":ti,ab,kw OR (Heart NEAR/1 surgery):ti,ab,kw) AND ("developmental disorder"/de OR "developmental delay"/de OR "Child Development"/de OR “developmental screening”/de OR "patient referral"/de OR "health care delivery”/de OR "Cognitive defect"/de OR "developmental language disorder "/de OR "Psychomotor Disorder"/de OR “early intervention”/de OR "early intervention":ti,ab,kw OR neurodevelopment*:ti,ab,kw) NOT (phenotype:ab,ti,kw OR gene:ab,ti,kw OR cell:ab,ti,kw OR mice:ab,ti,kw OR mouse:ab,ti,kw OR rat:ab,ti,kw OR rats:ab,ti,kw OR animal:ab,ti,kw)

CINAHL search string (16 August 2021 and 17 March 2023)

((MH Child+) OR (MH Infant+) OR (MH Adolescent) OR (TI child* OR AB child*) OR (TI infant* OR AB infant*) OR (TI famil* OR AB famil*) OR (TI *school OR AB *school) OR (TI p#ediatr* OR AB p#ediatr*) OR (TI prematur* OR AB prematur*)) AND ((MH "Heart Defects, Congenital") OR (TI "congenital heart disease*" OR AB "congenital heart disease*") OR (TI CHD OR AB CHD) OR (TI "congenital heart" OR AB "congenital heart") OR ((TI heart OR AB heart) N1 (TI surgery OR AB surgery))) AND ((MH "Child Development Disorders+") OR (MH "Developmental Disabilities") OR (MH "Child Development") OR (MH "Neurodevelopment") OR (MH "Referral and Consultation") OR (MH "Health Care Delivery") OR (MH "Cognition Disorders") OR (MH "Language Disorders") OR (MH "Psychomotor Disorders") OR (MH "Early Intervention+") OR (TI "early intervention" OR AB "early intervention") OR (TI neurodevelopment* OR AB neurodevelopment*)) NOT (TI ((phenotype OR gene OR cell OR mice OR mouse OR rat OR rats OR animal)) OR AB ((phenotype OR gene OR cell OR mice OR mouse OR rat OR rats OR animal))

Scopus search string (16 August 2021 and 17 March 2023)

((TITLE-ABS-KEY (neurodevelopmental) OR TITLE-ABS-KEY ("developmental delay*") OR TITLE-ABS-KEY ("developmental screen*") OR TITLE-ABS-KEY ("Cognition Disorder*") OR TITLE-ABS-KEY ("Language Disorder*") OR TITLE-ABS-KEY ("Psychomotor Disorder*") OR TITLE-ABS-KEY ("developmental Disorder*")) AND (((TITLE-ABS-KEY ("congenital heart")) OR (TITLE-ABS-KEY (heart/1 AND malformation)) OR (TITLE-ABS-KEY (heart/1 AND defect)) OR (TITLE-ABS-KEY (chd)) OR (TITLE-ABS-KEY (heart/1 AND surgery))) AND (TITLE-ABS-KEY (child* OR adolescen* OR infant* OR famil* OR *school OR pediatric* OR paediatic* OR prematur*)))) AND NOT (TITLE-ABS-KEY (phenotype OR gene OR cell OR mice OR mouse OR rat* OR animal)
