# Supplementary material for: Implementing neurodevelopmental follow‐up care for children with congenital heart disease: A scoping review with evidence mapping
Source: Dev Med Child Neurol. 2023 Jul 8;66(2):161–75. doi: 10.1111/dmcn.15698 (PMC10953404; doi:10.1111/dmcn.15698)
Supplement: Supplementary file 4 — Figure S2: Visual representation of the relationship between publications, clinical centres/sites, and care pathways. [file DMCN-66-161-s002.pdf]

Clinical centre/hospital	Publication	Care pathway
Herma Heart Centre	Soto et al	1
	Brosig et al	2
	Ruehl et al	3
Monroe Carell Jr. Children's Hospital	Chorna et al	4
University of Virginia Children's Hospital	Michael et al	5
Nationwide Children's Hospital (LAUNCH)	Davis et al	6
C.S Mott Children's Hospital	Loccoh et al	7
Children's Hospital Colorado	di Maria et al	8
Texas Children's Hospital (CDOP)	Monteiro et al	9
Utah's Heart Center	Glotzbach et al	10
Children's Healthcare of Atlanta	Tan et al	11
	Alam et al	12
Children's Hospital of Philadelphia (CKDP)	Favilla et al	13
Doernbecher Children's Hospital	Lee et al	14
Stollery Children's Hospital	Robertson et al	15
Hospital for Sick Children	Roberts et al	16
Sainte-Justine University Hospital (CINC)	Fourdain et al, '20	17
	Fourdain et al, '21	17
Queensland Children's Hospital	Eagleson et al	18, 19
	Eagleson et al*	19
Children's Hospital at Westmead	Quadir et al	20
Lille University Hospital	Domanski et al*	21, 22
	Domanski et al*	21, 22
