# Supplementary material for: Implementing neurodevelopmental follow‐up care for children with congenital heart disease: A scoping review with evidence mapping
Source: Dev Med Child Neurol. 2023 Jul 8;66(2):161–75. doi: 10.1111/dmcn.15698 (PMC10953404; doi:10.1111/dmcn.15698)
Supplement: Supplementary file 5 — Table S1: Keyword and MeSH terms for initial search strategy. [file DMCN-66-161-s001.pdf]

Table S1. Keyword and Mesh terms for initial search strategy

Population	Keyword terms	Mesh terms
**Age: Infants, children, youth**	child*[tiab] OR infant*[tiab] OR adolescen*[tiab] OR teenage*[tiab] OR p$ediatr*[tiab] OR teen*[tiab] OR youth[tiab] OR kids[tiab] OR newborn[tiab] OR famil*[tiab] OR neonat*[tiab] OR prematur*[tiab] OR $school[tiab]	“Child”[Mesh] OR “Infant”[Mesh] OR “Adolescent”[Mesh] OR Pediatrics[Mesh]
	**AND**	
**Condition: congenital heart disease +/- surgical intervention**	(congenital[tiab] AND heart disease*[tiab]) OR (congenital*[tiab] AND heart defect*[tiab]) OR heart abnormalit*[tiab] OR heart malformation*[tiab] OR heart disease*[tiab] OR cardiac abnormalit*[tiab] OR cardiac malformation*[tiab] OR cardiac diseas*[tiab] OR cardiac defect*[tiab] OR CHD [tiab]	“Heart Defects, Congenital”[Mesh] OR “fetal heart”[Mesh]

Concept of interest	Keyword terms	Mesh terms
**Models of care for neurodevelopmental assessment, screening, follow-up**	(neurodevelopment*[tiab] AND (evaluat* OR outcome* OR risk OR assess* OR test* OR follow-up OR delay* OR disabilit* OR disorder* OR impair* OR limit* OR problem* OR function* OR change* OR prognosis OR screen* OR examin* OR refer* OR tool* OR surveillance OR therap* OR intervent* OR program* OR model* OR care) [tiab]) OR early intervention[tiab] OR neuropsychological[tiab] OR neurocognit*[tiab] OR neurobehavi*[tiab] OR neuromotor*[tiab]OR neurologic*[tiab]	“Epidemiologic Studies”[Mesh] OR Referral and Consultation[Mesh] OR “delivery of health care”[Mesh] OR “quality of health care”[Mesh] OR “Outcome Assessment, Health Care”[Mesh] OR “Child Development”[Mesh] OR “Cognition Disorders”[Mesh] OR “Neurodevelopmental Disorders”[Mesh] OR “Neuropsychological Tests”[Mesh] OR “Intelligence Tests”[Mesh] OR “Mass screening”[Mesh]
