# Supplementary material for: Implementing neurodevelopmental follow‐up care for children with congenital heart disease: A scoping review with evidence mapping
Source: Dev Med Child Neurol. 2023 Jul 8;66(2):161–75. doi: 10.1111/dmcn.15698 (PMC10953404; doi:10.1111/dmcn.15698)
Supplement: Supplementary file 6 — Table S2: Characteristics of included studies reporting on implementation and/or evaluation of individual neurodevelopmental follow‐up programs and pathways. [file DMCN-66-161-s007.docx]

**Table S2:** Characteristics of included studies reporting on implementation and/or evaluation of individual neurodevelopmental follow-up programs and pathways

| **Study details** | | | | | **Program details** | **Participant/patient details** | | | | | | **Outcomes reported** | | | | |
| --- | --- | --- | --- | --- | --- | --- | --- | --- | --- | --- | --- | --- | --- | --- | --- | --- |
| *Author, Year ^reference^* | *Country* | *Focus/Aim* | *Study design, data type* | *Data collection timeframe* | *Clinic/program name and location* | *N* | *Male (n)* | *Mean age (range)* | *Diagnosis/clinical characteristics* | | | *Outcome type* | | | | |
|  |  |  |  |  |  |  |  |  | *Early open-heart surgery or cyanotic lesions* | *CHD with comorbidities* | *High risk~* | *Acceptability* | *Adoption* | *Effectiveness* | *Cost* | *Implementation factors* |
| Soto et al, 2011 ^27^ | USA | Implementation of developmental follow-up program and description of outcomes | Retrospective chart review, quantitative | July 2007 – June 2011 | Herma Heart Center Developmental Follow-Up Clinic, Children’s Wisconsin, Wisconsin | 95 | 54 | 7 months | SV/BV | - | - | Yes | Yes | - | - | Yes |
| Brosig et al, 2014 ^32^ | USA | Integration of outpatient psychologist services with developmental follow-up program | Retrospective chart review and prospective outcome measures with comparison to population norms, quantitative | July 2007 – June 2011 | Herma Heart Center Paediatric Cardiology Outpatients & Developmental Follow-Up Clinic, Children’s Wisconsin, Wisconsin | 79 | 59 | 100 (32-204) months | SV/BV | - | - | - | Yes | Yes | - | Yes |
| Ruehl et al, 2022 ^33^ | USA | Describe design and implementation of Educational Achievement Partnership Program to improve health and academic outcomes of children with CHD | Operational analyses via program database and family feedback survey, quantitative | 2015-2021 | Herma Heart Center Educational Achievement Partnership Program, Children’s Wisconsin, Wisconsin | 700+ | - | - | - | - | - | Yes | Yes | Yes | Yes | - |
| Chorna et al, 2016 ^34^ | USA | Develop and implement integrated care program for all children with CHD | Retrospective chart review, quantitative | January 2014 – January 2015 | CHD Follow-Up Program/ NICU Developmental Follow-Up Clinic, Monroe Carell Jr. Children's Hospital at Vanderbilt, Nashville | 132 | 57 | 9 months [median] | AVSD, AS, atresia, CoA, PA/critical PS, TOF, TGA, TAPVR, Truncus arteriosus, TA, HLHS | Yes | - | Yes | Yes | Yes | - | Yes |
| Michael et al, 2016 ^39^ | USA | Describe implementation of protocol for developmental follow-up of children with high-risk CHD at single institution | Retrospective chart review with prospective outcome measures and comparison to historical cohort at same clinic, quantitative | October 2013- October 2014 | University of Virginia Children’s Hospital Heart Center, Newark, New Jersey | 31 | 17 | - | SV/BV | - | - | - | - | Yes | - | - |
| Davis et al, 2018 ^40^ | USA | Describe development and implementation of a multidisciplinary program for patients and families affected by complex single-ventricle heart defects | Not clearly reported. Quality improvement processes including statistical process charts. | 2014-2016 | Lifetime Strategies and Outcomes for Single Ventricle and Complex Hearts (LAUNCH), Nationwide Children’s Hospital, Columbus, Ohio | - | - | - | SV | - | - | - | Yes | - | - | Yes |
| Loccoh et al, 2018 ^42^ | USA | Determine prevalence and patterns of neurodevelopmental evaluation at neurodevelopmental follow-up clinic after infant cardiac surgery | Retrospective electronic chart review, quantitative | April 2011-March 2014 | Neurodevelopmental Follow-up Clinic at University of Michigan C.S Mott Children’s Hospital, Ann Arbor, Michigan | 94 | 52 | 12 (10-49) months | Early OHS | - | - | - | Yes | - | - | Yes |
| Di Maria et al, 2019 ^36^ | USA | Evaluate multidisciplinary approach to surveillance-based care pathway for children after Fontan operation | Audit and quality improvement processes including statistical process charts, quantitative | January 2016-September 2017 | Fontan Multidisciplinary Clinic, Children’s Hospital Colorado, Colorado | 86 | 46 | - | SV, HLHS | - | - | - | Yes | Yes | - | Yes |
| Monteiro et al, 2019 ^44^ | USA | Describe prevalence and patterns of referral to outcomes clinic of patients who underwent surgery for CHD during infancy | Retrospective electronic database review, quantitative | April 2013 – May 2017 | The Cardiac Developmental Outcomes Program (CDOP) clinic, Texas Children’s Hospital, Texas | 244 | 142 | 7 months | Early OHS | - | - | - | - | Yes | - | - |
| Glotzbach et al, 2020 ^38^ | USA | Describe referral patterns and outcomes of neurodevelopmental program for high-risk children with CHD | Retrospective electronic chart review, quantitative | July 2015-December 2017 | Utah’s Heart Center Neurodevelopmental Program (HCNP), University of Utah and Primary Children's Hospital, Salt Lake City | 148 | 61 | - | AVSD, CoA, TOF, TGA, TAPVR, TA, Truncus arteriosus, DILV, DORV, HLHS, early OHS | Yes | - | - | Yes | - | - | Yes |
| Tan et al, 2020 ^47^ | USA | Identify the frequency of children with CHD considered at risk for autism at a single cardiac neurodevelopmental program and highlight opportunities for improvement | Retrospective electronic chart review, quantitative | February 2018-February 2019 | Children’s Healthcare of Atlanta Cardiac Neurodevelopmental Program, Children’s Healthcare of Atlanta, Atlanta | 134 | 86 | 108 (36-228) months | - | - | Yes | - | - | Yes | - | - |
| Alam et al, 2022 ^35^ | USA | Evaluate impact of neurodevelopmental evaluation on service use by school-aged children in a program supporting children with single ventricle CHD | Retrospective chart review and phone survey including clinical comparison group, quantitative and qualitative | 2012-2018 | Children’s Healthcare of Atlanta Cardiac Neurodevelopmental Program, Children’s Healthcare of Atlanta, Atlanta | 29 | 11 | 108 (60-156) months | PA/IVS, TA, DILV, DORV, HLHS, Ebstein’s anomaly | Yes | - | Yes | - | Yes | - | Yes |
| Favilla et al, 2021^48^ | USA | Identify factors associated with neurodevelopment and referral for early intervention for children with repaired Tetralogy of Fallot | Retrospective chart/registry audit, quantitative | 2012-2018 | Cardiac Kids Developmental Follow-up Program (CKDP), Children’s Hospital of Philadelphia, Philadelphia | 49 | 33 | 4 months at first visit, 13 months at second visit [median] | TOF | - | - | - | - | Yes | - | - |
| Lee et al, 2021 ^49^ | USA | Evaluate referral rates for early intervention and neurodevelopmental services, and outcomes in patients undergoing congenital heart surgery in the first year of life | Retrospective electronic chart review, quantitative and qualitative | January 2013-December 2018 | Neurodevelopmental Service, Doernbecher Children's Hospital, Portland, Oregon | 60 | 37 | 8 (2-77) months [median] | TOF, TGA, TAPVR, HLHS, HRHS | Yes | - | - | Yes | Yes | - | - |
| Robertson et al, 2011 ^50^ | Canada | Present an interprovincial longitudinal neurodevelopmental follow-up program for children after complex cardiac surgery | Retrospective review of database/registry, quantitative | 1996-2010 | Registry and Follow-up of Complex Pediatric Therapies Program of Western Canada, Hub at Stollery Children's Hospital, Edmonton, Alberta | 659 | - | - | PA, TOF, TGA, TAPVR,, Truncus arteriosus, DORV, IAA, HLHS, other, early OHS | Yes | - | - | Yes | Yes | - | - |
| Roberts et al, 2021^56^ | Canada | Describe the implementation and uptake of a Cardiac Neurodevelopment Program; examine parental mental health and relationship to child outcomes | Cross sectional study of subset of children enrolled in program. Quantitative, chart review and clinical measures. | June 2017-January 2019 | Cardiac Neurodevelopment Program at the Hospital for Sick Children, Toronto, Ontario | 240 | 120 | IQR 3-41 months | TOF, TGA, SV, CoA, Truncus arteriosus, IAA, Ebstein, TAPVR | - | - | - | Yes | Yes | - | - |
| Fourdain et al, 2020 ^51^ | Canada | Evaluate an early systematic interdisciplinary developmental follow-up and individualized intervention program for children with complex surgical congenital heart disease | Prospective longitudinal cohort and historical comparison cohort | June 2016 | Clinique d’Investigation Neurocardiaque (CINC), Sainte-Justine University Hospital Center, Montreal, Québec | 41 | 17 | 44 months | CoA, TOF, TGA, | - | - | - | Yes | Yes | - | - |
| Fourdain et al, 2021 ^52^ | Canada | Describe developmental trajectory and physical therapy interventions received by infants with CHD enrolled in systematic developmental follow-up program | Retrospective chart review, quantitative | Mar 2013-Jun 2016 | Clinique d’Investigation Neurocardiaque (CINC), Sainte-Justine University Hospital Center, Montreal, Québec | 29 | 19 | - | VSD/AVSD, TGA, DORV | - | - | - | - | Yes | - | - |
| Eagleson et al, 2020 ^53, 54*^ | Australia | Describe experience establishing a neurodevelopmental follow up program for neonates with complex congenital heart disease and extracorporeal life support survivors | Retrospective chart review/registry audit, parental survey, quantitative | 2013-2015 | Queensland Paediatric Cardiac Service neurodevelopmental long-term follow-up programme, Queensland Children’s Hospital, Brisbane | 55 | - | - | TGA, Norwood procedure | Yes | - | Yes | Yes | - | - | Yes |
|  |  |  | Retrospective chart review/registry audit, quantitative | May 2018- Dec 2019 | CHD LIFE (Long-term Improvement in Functional hEalth), State-wide across Queensland | 163 | - | - | Early OHS | - | - | - | - | Yes | - | Yes |
| Quadir et al. 2022^31^ | Australia | Highlight importance of neurodevelopmental follow-up for cohort of infants surviving ECMO therapy | Retrospective chart review/registry audit, quantitative | 2008-2018 | Children's Hospital at Westmead's Grace neonatal development clinic | 37 | 23 | - | HLHS, SV, TGA, critical aortic stenois, Truncus arteriosus, DORV, TOF, Ebstein’s, PS, AVSD, VSD | - | - | - | Yes | - | - | - |
| Domanski et al, 2022^28, 29^* | France | Report implementation of new structured neurodevelopmental follow-up program at institution | Not clear (likely retrospective review of database), quantitative | October 2020- May 2022 | KidsHearts, Lille University Hospital, Lille, France | 64 | - | 10 (5-15) months | Early OHS before 12 months of age | - | - | Yes | Yes | - | - | - |

* denotes conference abstract; ~ as defined/categorised in American Heart Association guidelines ^2^; AVSD, atrioventricular septal defect; CHD, Congenital Heart Disease; CoA, coarctation of the aorta; DILV, double inlet left ventricle; DORV, double outlet right ventricle; ECMO, extracorporeal membrane oxygenation; HLHS, hypoplastic left heart syndrome; HRHS, hypoplastic right heart syndrome; IAA, interrupted aortic arch; IQR, interquartile range; PA/IVS, pulmonary atresia/intact ventricular septum; PS, pulmonary stenosis; TOF, Tetralogy of Fallot; TGA, transposition of the great arteries; TAPVR, total anomalous pulmonary venous return/connection; TA, tricuspid atresia; VSD, ventricular septal defect; SV, single ventricle (not other specified); BV, biventricular (not other specified); OHS, open heart surgery. Comorbidities include: organ transplant, extracorporeal membrane oxygenation, left ventricular assist device, genetic diagnosis, cardiopulmonary resuscitation.
