# Supplementary material for: Implementing neurodevelopmental follow‐up care for children with congenital heart disease: A scoping review with evidence mapping
Source: Dev Med Child Neurol. 2023 Jul 8;66(2):161–75. doi: 10.1111/dmcn.15698 (PMC10953404; doi:10.1111/dmcn.15698)
Supplement: Supplementary file 7 — Table S3: Characteristics of included studies reporting on surveys or overviews of neurodevelopmental follow‐up practices. [file DMCN-66-161-s006.docx]

Table S3: Characteristics of included studies reporting on surveys or overviews of neurodevelopmental follow-up practices

| **Study details** | | | | | **Site/participant details** | | | | **Outcomes reported** | |
| --- | --- | --- | --- | --- | --- | --- | --- | --- | --- | --- |
| *Author, Year ^reference^* | *Country* | *Focus/Aim* | *Data collection* | *Design* | *N (sites or individuals)* | *Region, Country* | *Site type* | *Role of survey participants* | *Key message* | *Gaps/opportunities* |
| Knutson et al, 2016 ^9^ | USA | Identify developmental interventions, guideline adherence, and barriers and enablers to developmental support of children with CHD | February 2014 – June 2014 | Survey | 326 individuals | Minnesota, USA | Private practice, community clinic, academic, Health Maintenance Organisation | Family physicians, general paediatricians | Lack of awareness about AHA guidelines and risks of developmental problems in children with CHD, particularly in primary care providers | Greater education of primary care providers and collaboration with paediatric cardiology required to improve outcomes |
| Di Maria et al, 2019 ^37^ | USA | Identify practice patterns in surveillance testing within programs caring for Fontan patients in the USA | - | Survey | 11 sites | USA | Academic centre (n=6); clinical centre (n=5) | Physicians | Significant practice variability in type and timing of surveillance testing | - |
| Miller et al, 2020 ^41^ | USA | Identify the characteristics and practices of neurodevelopmental follow-up approaches of CNOC member centres | November 2017 – May 2018 | Survey | 23 sites | USA (n=20), Canada (n=2), Europe (n=1) | - | Intensivists, nurses, psychologists, cardiologists, neurologists, developmental paediatricians, quality manager | Although practices vary greatly, most centres focus on younger ages using common, standardised assessment measures | Gap at school-age and beyond; limited understanding of relative importance of components on outcomes |
| Sidhu et al, 2022 ^55^ | USA | Evaluate the awareness and adherence of paediatric cardiologists to AHA guidelines relating to developmental referral and surveillance of children CHD | - | Survey | 129 individuals | USA | Mostly hospital-based | Paediatric cardiologists | Most respondents worked at centres with formal neurodevelopmental follow-up programs. Over half lacked familiarity with AHA guidelines. Underutilisation of developmental surveillance testing by primary care physicians. | Greater collaboration between primary care providers and paediatric cardiologists required. Incorporate developmental surveillance and screening recommendations into AAP guidelines. |
| Basile et al, 2022^13^ | USA | Identify practices for neurodevelopmental, psychosocial, and transitional outpatient care for children with CHD in North American hospitals | June 2019 | Survey | 48 sites | USA | Hospital-based paediatric cardiac clinics | Nurses, psychologists, cardiologists | Care practices are highly varied across the cardiac clinics represented, and only small proportion able to meet AHA guidelines due to implementation challenges | Improve the neurodevelopmental management of school-aged and adolescent children with CHD |
| Kasparian et al^46^, 2022 | USA and Canada | Quantify implementation of telehealth  practices in neurodevelopmental follow-up programs at CNOC member centres during COVID-19 | July 2020- August 2020 | Survey | 30 sites | USA (n=29)  Canada (n=1) | Medical institutions that  provide care for children with CHD | Psychologists, physicians, nurses, developmental specialists | 24/30 sites successfully transitioned to delivering some (but not all) elements of neurodevelopmental follow-up virtually. Challenges with technology, providing equitable care, and provider perceptions need to be addressed. | Research is required about validity and practical administration of standardised  psychological and neurodevelopmental assessments for children over telehealth. An opportunity exists to provide training and guidance in telehealth use for follow-up clinics. |
| Leon et al, 2022^45^ | USA | Determine variation in practice for neonatal CHD care within a network of high-volume neonatal cardiac surgery programs in North America | April 2021- September 2021 | Survey | 29 sites | USA and Canada (not specified how many from each region) | Children’s Hospital’s Neonatal Consortium member hospitals (sites with Level IV NICUs) | - | Neurodevelopmental follow-up care is provided in almost all centres (97%). However, significant variations exist in how this care is delivered and integrated into cardiology, NICU and other clinical practices and pathways. | Research needs to understand role of neurodevelopmental testing in patients with CHD. Standards for postnatal CHD care will decrease inter-centre variability. |
| Bolduc et al, 2022 ^15^ | Canada | Describe current developmental follow-up practices and challenges in centres performing paediatric open-heart surgery in Canada | - | Survey and telephone interviews | 8 sites | Canada, across Alberta, British Columbia, Nova Scotia, Ontario, Quebec | Paediatric hospital, university health centre, general hospital | Nurses, psychologists, cardiologists, paediatricians, neonatologists | Current practice is varied and sub-optimal. Strict eligibility criteria for access. | Sites would benefit from a more systematic approach. Develop national recommendations to optimize the developmental follow-up practices in Canada. |
| Hoskote et al, 2021 ^43^ | UK | To describe neurodevelopment and follow-up practices in preschool children with heart disease in London | January 2014- January 2015 | Secondary analysis of cross-sectional convenience sample | 3 sites | London, UK | Tertiary children’s cardiac centres | - | Variability in services received. Many children, despite being in the high-risk category, did not appear to be under appropriate services for their developmental needs. | No UK-based guideline. Structured neurodevelopmental follow-up programs needed. |
| Smith et al, 2019 ^14^ | South Africa | Determine neurodevelopmental screening and referral practices for children with CHD in South Africa | - | Survey | 20 sites | South Africa across Free State and Northern Cape provinces | Academic hospital, public hospital, private sector | Cardiac surgeons, cardiologists, paediatricians | Low awareness of AHA guideline. Current practice is varied and sub-optimal. | A national survey in a larger sample of cardiac care practitioners is needed. |
| Feldmann et al, 2022 ^16^ | Europe | Information about the implementation of neurodevelopmental follow-up programs across Europe | April 2019- June 2020 | Survey | 25 sites | Europe including UK, Spain, France, Netherlands, Germany, Switzerland, Hungary, Slovenia, Italy, Austria, Latvia | - | Intensivists, cardiac surgeons, cardiologists, neurologists, developmental paediatricians, neonatologists, paediatric radiologists | Two-thirds of centres have or want a neurodevelopmental program. Interested in joining a European neurodevelopmental outcome registry but current data collection rates low (32%). | Adaptation of guidelines to context needed. Programs could benefit from standardization and systematic recording by creating a European outcome register. |
| Natterer et al, 2022^30^ | Switzerland | Describe implementation of a national registry for documentation of neurodevelopmental follow-up and outcomes at all national centres | 2019-2021 | Implementation framework | 4 paediatric heart centres, 16 follow-up sites | Heart centres in Zurich, Bern, Lausanne and Geneva. Follow-up sites nationwide across Switzerland | Sites performing intervention for children with CHD in first 6 weeks of life | Paediatric cardiology, cardiac surgery, paediatric in-tensive care, paediatric anesthesia, and developmental paediatrics | Describes neurodevelopmental follow-up procedures at Swiss centres and successful implementation of nationwide registry capturing information for 138 children. Integration of both the registry and ND follow-up within an existing network/process for follow-up of high-risk infants was beneficial. | Registry provides foundation for national collaboration and standardisation of practices, as well as enabling ongoing research projects. |

AAP, American Academy of Paediatrics; AHA, American Heart Association; CHD, congenital heart disease; CNOC, Cardiac Neurodevelopmental Outcome Collaborative; NICU, Neonatal Intensive Care Unit; UK, United Kingdom; USA, United States of America
