# Supplementary material for: Implementing neurodevelopmental follow‐up care for children with congenital heart disease: A scoping review with evidence mapping
Source: Dev Med Child Neurol. 2023 Jul 8;66(2):161–75. doi: 10.1111/dmcn.15698 (PMC10953404; doi:10.1111/dmcn.15698)
Supplement: Supplementary file 8 — Table S4: Detailed descriptions of programs and care pathways for neurodevelopmental follow‐up of children with CHD reported in included studies. [file DMCN-66-161-s008.docx]

|  | Service/site details | | | | | Program/pathway care elements and processes | | | | | | |
| --- | --- | --- | --- | --- | --- | --- | --- | --- | --- | --- | --- | --- |
| *Number, Author ^reference^* | *Program/ pathway name, location* | *Year*  *established* | *Care setting type* | *Service structure* | *Eligibility* | | *Referral to program or pathway* | *Providers* | *Overview of follow-up processes and domains* | *Frequency or timing of follow-up* | *Referral out to intervention/ other services* | *Standardised assessment tools reported* |
| 1, Soto et al ^1^ | Herma Heart Center Developmental Follow-Up Clinic, Children’s Wisconsin, Wisconsin USA | 2007 | Children’s Hospital | Centralised | Children < 3 years with complex CHD who undergo surgery within first 30 days of life, or at risk of developmental problems | | Cardiologists, nurse practitioners, and therapists identify eligible patients before discharge. Families given information and referrals made to program co-ordinator who contacts families and schedules appointments. | MD clinic: nurse, general/ developmental paediatrician, speech therapist, occupational therapist, and physical therapist | Medical and developmental history; demographic information; physical exam; ND screening | Every 6 months, from 6 months to 3 years old | To other specialists or appropriate therapy as required. To clinic developmental psychologist if needed. | 1. Bayley Scales of Infant and Toddler Development |
| 2, Brosig et al ^2^ | Herma Heart Center Paediatric Cardiology Outpatients co-located Psychologist, Children’s Wisconsin, Wisconsin USA | 2007 | Children’s Hospital | Centralised | Children with CHD undergoing follow-up in outpatient cardiology clinics who were flagged to have behavioural health concerns | | Cardiologists and nurses ask about the child’s emotional, behavioural, and academic functioning in outpatient clinic and referred to psychologist if needed | Co-located clinical psychologist in Cardiology Outpatients | Medical history; functioning/ psychological assessment | *-* | *-* | 1. Child Behaviour Checklist  2. Pediatric Quality of Life Inventory™ |
| 3, Ruehl et al ^3^ | Educational Achievement Partnership Program (Herma Heart Center Developmental Follow-Up Clinic) Children’s Wisconsin, Wisconsin USA | 2015 | School and children’s hospital | Centralised with local links between school, family, community, and hospital | Children with congenital and acquired heart disease | | By child’s medical provider | Hospital-based school liaison (educator) plus MD clinic: nurse, general/ developmental paediatrician, speech therapist, occupational therapist, and physical therapist | Medical, neuropsychological, and developmental history; demographic information; physical exam; formal assessment across developmental, school, psychosocial and academic domains | As needed to achieve educational goals then transition into annual follow-up | Plan and letters sent to schools for cardiac health and educational supports. Guidebook and presentation for schools. | Not reported |
| 4, Chorna et al ^4^ | CHD Follow-Up Program/ NICU Developmental Follow-Up Clinic, Monroe Carell Jr. Children's Hospital at Vanderbilt, Nashville | 2014 | Children’s Hospital | Centralised with phone calls (100-mile catchment) | Any patient meeting AHA guideline criteria for early developmental follow-up* rather than specific diagnoses | | Children <2 years of age who had been discharged from the hospital and met eligibility criteria flagged, and Cardiology discharge planners made referrals to clinic coordinator | Occupational therapist, physical therapist, paediatric nurse practitioner, orthotist, psychologist and developmental specialist | At 6-12 months: interview with trained developmental tester via phone; at 18 months full developmental history; neurological examination and assessment | Every 6-12 months, until 3 years old, depending on concerns | Referrals made to early intervention and formal developmental and medical evaluation services at time of visit as needed | 1. Bayley Scales of Infant and Toddler Development  2. Ages and Stages Questionnaire  3. Child Behaviour Checklist |
| 5, Michael et al ^5^ | University of Virginia Children’s Hospital Heart Center, Newark, New Jersey | 2013 | Children’s Hospital | Centralised but wide distribution of patients | Children who had undergone cardiac surgery within the first year of life and meet AHA 2012 guideline criteria for early developmental follow-up* | | Integrated into postoperative pathway. Patients identified at time of surgical procedure; inpatient evaluation and family education by developmental paediatrics team before discharge; follow-up scheduled; phone call reminders | Inpatient evaluation: physician, nurse practitioner | Return for formal developmental outpatient follow-up (no further details provided) | At 6 months (or 3 months if concern for neurologic injury) | Not reported | Not reported |
| 6, Davis et al ^6^ | Lifetime Strategies and Outcomes for Single Ventricle and Complex Hearts (LAUNCH), Nationwide Children’s Hospital, Columbus, Ohio | 2013 | Children’s Hospital | Centralised | Children with CHD and single-ventricle defects | | Consults initiated on admission after birth | Physical therapist, occupational therapist, speech therapist, nurse care coordinator and single-ventricle nurse practitioner plus social worker and psychologist via rest of pathway | Neurodevelopmental testing wrapped in broader care pathway including foetal preparation, medical management and psychosocial care | At birth, 3 months, 12 months, 18 months, and 24 months | Before discharge, referrals placed to early intervention program as needed. Referred to the appropriate intervention specialist based on screening results. | Not reported |
| 7, Loccoh et al ^7^ | ND Follow-up Clinic at University of Michigan C.S Mott Children’s Hospital, Ann Arbor, Michigan | 2011 | Children’s Hospital | Centralised | Children who have undergone cardiac surgery within the first year of life | | All infants who have cardiac surgery < 12 months old at the hospital referred to the clinic; before or after discharge the clinic contacts parents to schedule appointment | Paediatric psychologist, paediatric nurse practitioner, social worker, and dietician | Neurodevelopmental evaluation; review of parent-reported measures of development and psychosocial functioning; physical exam (neuromotor assessment) | At 9-12 months, 18-12 months, and 3 years | Referrals are made to local early intervention or private programs for physical, occupational, or speech therapies | 1. Bayley Scales of Infant and Toddler Development |
| 8, di Maria et al ^8^ | Fontan Multidisciplinary Clinic, Children’s Hospital Colorado, Colorado | 2016 | Children’s Hospital | Centralised with 7-state catchment area | Children with single ventricle heart disease who had undergone Fontan operation | | Referred to MD clinic by Cardiology, where seen by paediatric neuropsychologist for screening who could then refer for formal testing | Nurse coordinator, cardiologist, pulmonologist, hepatologist, neuropsychologist, paediatric psychologist, dietician, and psychosocial wellness team (social worker, chaplain, child life specialist) | Medical history; Health‐Related Quality of Life questionnaire; screening of emotional and behavioural functioning; neuropsychological interview (including neurobehavioral examination, and parent‐report rating measures of cognition and learning plus a screening questionnaire for those >4 years old); other testing* | Every 2 years from 5 – 18 years old | Not reported | 1. Pediatric Quality of Life Inventory™  2. Behavior Assessment System for Children-3 |
| 9, Monteiro et al ^9^ | The Cardiac Developmental Outcomes Program (CDOP) clinic, Texas Children’s Hospital, Texas | 2013 | Children’s Hospital | Centralised | Infants who have undergone surgery and transcatheter interventions for CHD during the first 3 months of life, as well as some children and adolescents with CHD and ND concerns | | All patients are identified and have inpatient consultation by developmental behavioural paediatrician prior to discharge; called and scheduled for follow-up. Also, external referrals. | Bilingual (English and Spanish) clinic coordinator. Developmental behavioural paediatricians and child psychologists | Medical history; demographics; comprehensive clinical and neurological examination; standardised developmental testing | At 6 months, 12 months. 18 months and 24 months. Yearly or as clinically indicated thereafter. | Referrals for early intervention programs, private therapies (speech, occupational, and/or physical) and other medical services made during clinic visits | 1. Cognitive Adaptive Test/Clinical Linguistic and Auditory Milestone Scale |
| 10, Glotzbach et al ^10^ | Utah’s Heart Center Neurodevelopmental Program (HCNP), University of Utah and Primary Children's Hospital, Salt Lake City | 2015 | Children’s Hospital | Centralised | Children who meet AHA guideline criteria for early developmental follow-up* | | From primary care providers, cardiologists and self-referrals. In late 2015 referrals at time of discharge from neonatal CHD surgery. | Developmental paediatrician, paediatric psychologist, speech, occupational and physical therapists, and paediatric cardiologist | Screening and evaluation (not specified) | At 12–24 months, 4–6 years, and 8–14 years | Referral to the University of Utah Developmental Assessment Clinics for a comprehensive neuropsych evaluation. Referrals to speech therapy and local early intervention program enrolment. | Not reported |
| 11, Tan et al ^11^ | Children’s Healthcare of Atlanta Cardiac ND Program, Children’s Healthcare of Atlanta, Atlanta | 2018 | Children's healthcare system including 3 hospitals, community, and urgent care centres in Metro Atlanta | Centralised | Children who meet AHA guideline criteria for early developmental follow-up* | | Not reported | Cardiologist, neurologists, psychologist, clinical neuropsychologist | Flexible neuropsychological evaluation including medical review; behavioural observations; developmental history via parent interview; and ND assessment | Not reported | Referred to an autism specialist after completion of the evaluation | 1. Behavior Assessment System for Children-3  2. Adaptive Behaviour Assessment System |
| 12, Alam et al ^12^ | Children’s Healthcare of Atlanta Cardiac ND Program, Children’s Healthcare of Atlanta, Atlanta | 2012 | Children's healthcare system including 3 hospitals, community, and urgent care centres in Metro Atlanta | Centralised | Children with single ventricle CHD who had also undergone a Fontan operation | | Not reported | Cardiologist, neurologist, psychologist, clinical neuropsychologist | 3 appointments: 1. review of medical and school records; 2. caregiver interview; 3. ND assessment using standardised tests and parent/teacher rating scales; meeting with family for results and referrals | Not reported | Referrals to other specialists and early intervention services as needed | Not reported |
| 13, Favilla et al ^13^ | Cardiac Kids Developmental Follow-up Program (CKDP), Children’s Hospital of Philadelphia, Philadelphia | 2012 | Children’s Hospital | Centralised | Catheter-based or surgical intervention within the first 6 months of life, or neonatal hospitalization > 10 days | | Not reported | Paediatrician, clinical psychologist, occupational therapist and physical therapist | Interviews with parents; standardised assessments | At least once per year | Referral to early intervention by caregiver or provider | 1. Bayley Scales of Infant and Toddler Development  2. Bayley Infant ND Screener  3. Peabody Developmental Motor Scale |
| 14, Lee at al ^14^ | ND service, Doernbecher Children's Hospital, Portland, Oregon | 2013 | Children’s Hospital | Centralised but some children had evaluations, follow-up, referral, and intervention performed at facilities closer to home | Children with congenital heart disease who had cardiac surgery within the first year of life | | Referred by cardiologists, neurologists, neonatal or paediatric intensivists, and primary care providers | No reported | Neurodevelopmental evaluation (but not standardised and differed based on provider and visit) | Not reported | Referred to early intervention after evaluation or directly by providers | 1. Bayley Scales of Infant and Toddler Development  2. Ages and Stages Questionnaire  3. Cognitive Adaptive Test/Clinical Linguistic and Auditory Milestone Scale  4. Preschool Language Scales  5. Rossetti Infant-Toddler Language Scale  6. Alberta Infant Motor Scale  7. Peabody Developmental Motor Scale |
| 15, Robertson et al ^15^ | Registry and Follow-up of Complex Pediatric Therapies Program of Western Canada, Hub at Stollery Children's Hospital, Edmonton, Alberta | 1999, but widescale funding since 2006 | Hub at Children's Hospital but serves western provinces and northern territories of Canada | Decentralised with program partners in Alberta and across western Canada. Steering committee with sub-committees for data, outcome measurement and evaluation. | Complex cardiac surgery performed at age ≤ 6 weeks of age | | Referred by attending physician at participating acute care departments. Nurse coordinator registers child and discusses follow-up procedures with the parents. Contact is made with the follow-up clinic at the child’s tertiary centre. | Program membership: representatives from cardiology, cardiovascular surgery, nephrology, hepatology/ gastroenterology, and neonatal and paediatric intensive care.  Plus, at follow-up clinics: paediatrician with expertise in neurodevelopmental follow-up, nurse, physical therapist, psychologist, speech-language pathologist, and audiologists. | Paediatrician and nurse complete questionnaire about illnesses and hospitalizations as well as diet, feeding, sleep, immunisations, and parent support; record growth; perform medical and neurological examination; occupational therapist, dietician, or social worker assess child/family as necessary. | At birth, 3 months, 6-8 months, 18-24 months, 4-6 years and 8 years | Therapy and early developmental intervention as needed is available from time of hospital discharge at each site. | 1. Bayley Scales of Infant and Toddler Development  2. NEPSY  3. Behavior Rating Inventory of Executive Function Parent Form  4. Pediatric Quality of Life Inventory™  5. Child Behaviour Checklist  6. Behavior Assessment System for Children-3  7. Adaptive Behaviour Assessment System  8. Strength and Difficulties Questionnaire  9 Wechsler Preschool and Primary Scale of Intelligence  10. Wechsler Individual Achievement Test  11. Clinical Evaluation of Language Fundamentals  12. Child and Adolescent Scale of Participation  13. Visual Motor Integration |
| 16, Roberts et al ^16^ | Cardiac neurodevelopment program, Hospital for Sick Children, Toronto | 2014 | Children’s Hospital | Centralised | Children with congenital heart disease who had cardiac surgery on cardiopulmonary bypass within the first six weeks of life; infants with single ventricle pathology; infants with CHD born pre-term; infants with CHD and brain injury. | | Identified in hospital. Developmental counselling prior to discharge. | Collaborative program between Cardiology, Neonatology, Neurology and Psychology. Nurse practitioner. | Care coordination and continuity; counselling surrounding long-term neurodevelopmental outcomes; provide education and resources; Neonatal Neurodevelopmental Follow-Up Clinic assessment and intervention; Psychology assessments from 4 years old. | Cardiac Neurodevelopmental Follow-up Clinic: at 8 months, 12 months, 18 months and 36 months.  Neuropsychological assessment: 5 years, 8-9 years, 13-14 years, and 17-18 years. | Facilitate referral to community services. | 1. Bayley Scales of Infant and Toddler Development |
| 17, Fourdain et al ^17, 18^ | Clinique d’Investigation Neurocardiaque (CINC), Sainte-Justine University Hospital Center, Montreal, Québec | 2013 | Children’s Hospital | Centralised | Children presenting with moderate to severe CHD requiring cardiac surgery | | Children meeting the criteria at the hospital are referred at birth to the interdisciplinary neurocardiac clinic | All team contribute to clinical meetings but participation across each assessment varies: nurse practitioner coordinator, paediatric cardiac surgeon, paediatric neurologist, cardiologist, developmental paediatrician, physical therapist, occupational therapist, psychologist, speech-language pathologist, and nutritionist | Standard: neurological and physical exam; motor and cognitive assessments; socio-affective and behavioural screenings using parental questionnaires; physical therapy; parental education and support  Additional individualised intervention sessions: speech therapy; physical therapy; occupational therapy | Systematic age-based assessments: 4 months, 12 months, 24 months, 42 months.  Additional assessment timepoints as required based on screening results: 8 months, 18 months, 30 months | Individualized recommendati-ons to parents for educational activities, daily home exercises, or referral for direct intervention with a therapist | 1. Bayley Scales of Infant and Toddler Development  2. Ages and Stages Questionnaire  3. Child Behaviour Checklist  4. Behavior Assessment System for Children-3  5. Alberta Infant Motor Scale  6. Wechsler Preschool and Primary Scale of Intelligence  7. MacArthur–Bates Communicative Development Inventories  8. Modified Checklist for Autism in Toddlers  9. Sensory Profile |
| 18, Eagleson et al ^19^ | Queensland Paediatric Cardiac Service ND long-term follow-up programme, Queensland Children’s Hospital, Brisbane | 2013 | Children’s Hospital | Centralised | Infants who had undergone an Arterial Switch Operation, Norwood procedure or survived extracorporeal life support | | Parents provided written information and enrolled in program before discharge | MD allied health team and developmental paediatrician | Medical history; demographics; developmental paediatrician review; quality of life assessment; parental stress assessment; age-appropriate developmental assessment. Written report sent to families and clinicians involved in child’s care. | At 6 months, 12 months, 2 years, 4 years, 8 years, 11 years, 16 years | Therapeutic consultation and referrals to local providers for intervention | 1. Bayley Scales of Infant and Toddler Development  2. Pediatric Quality of Life Inventory™  3. Parenting Stress Index Short Form |
| 19, Eagleson et al ^19, 20^* | CHD LIFE (Long-term Improvement in Functional hEalth), State-wide across Queensland | 2018 | Various services across state including primary health care, community child development services, indigenous health services, and tertiary hospitals | Decentralised service delivery with an integrated, state-wide approach. | Infants who have open heart surgery < 12 months old | | During hospitalisation parents receive CHD, developmental and targeted care pathway education from allied health clinicians and advanced practice nurse. Referral to child’s local health service providers for follow-up. | Program leadership: MD, cross-divisional and inclusive of the consumer's voice.  Pathway: General practitioner, child health service, indigenous health service, cardiologist, paediatrician, and MD allied health providers via paediatric hospital outpatients, child development services, paediatric hospital, or private practice. | Reminders sent before key developmental time points. Families access/discuss screening or assessment with services they are using in their community for general and developmental paediatric review.  Local services provide secondary level screening; health check; family support. Age-appropriate developmental assessment with tools dependent on local availability and routine care. | At 6 months, 12 months, 18 months, 2.5-3.5 years, 4-5 years, 11-12 years, and 15 years | Intervention as indicated accessed via hospital, child development service, disability scheme, or private practice | 1. Bayley Scales of Infant and Toddler Development  2. Ages and Stages Questionnaire  3. Parents' Evaluation of Developmental Status Infant Scales  4. Behaviour Related Inventory of Executive Functioning  5. Child Behaviour Checklist  6. Adaptive Behaviour Assessment System  7. Wechsler Preschool and Primary Scale of Intelligence  8. Wechsler Individual Achievement Test  9. Woodcock Johnson Test of Achievement  10 Clinical Evaluation of Language Fundamentals  11. Sutherland Phonological Awareness Test  12. Children’s Memory Scale  13. Youth Self Report  14. Conners Rating Scales  15. Test of Everyday Attention for Children |
| 20. Quadir et al^21^ | Grace neonatal developmental clinic, Children's Hospital at Westmead, Sydney | - | Children’s Hospital | Centralised | Infants with CHD who have early open heart surgery | | Identified before discharge from neonatal intensive care unit | Multidisciplinary clinic | Neurodevelopmental evaluation (not further specified) | At 3–4 months, 1 year and 3 years | Not reported | 1. Bayley Scales of Infant and Toddler Development |
| 21. and 22. Domanski et al ^22, 23^ | KidsHearts, Lille University Hospital, Lille | 2020 | University Hospital Centre | Not clear (may have elements of both centralisation and decentralisation) | Infants who have open heart surgery < 12 months old | | Identified before surgery and enrolled prior to discharge into one of three pathways based on risk. | Neurologist to provide clinical evaluation before discharge. | Pathway 21: general paediatrician follow-up with parental neurodevelopmental questionnaire at key ages  Pathway 22: specialised centre-based follow-up  Third pathway in study was systematic evaluation 3 months after surgery to reassess pathway selection (not included as care pathway in review as more of a holding pathway) | At 3 months, 6 months and 12 months | Not reported | 1. Ages and Stages Questionnaire |

CHD, congenital heart disease; ND, neurodevelopmental; NICU, neonatal intensive care unit; MD, multidisciplinary; USA, United States of America.

**References**

1. Soto CB, Olude O, Hoffmann RG, et al. Implementation of a routine developmental follow‐up program for children with congenital heart disease: Early results. *Congenital heart disease* 2011; 6: 451-460.

2. Brosig C, Yang K, Hoffmann RG, et al. The role of psychology in a pediatric outpatient cardiology setting: preliminary results from a new clinical program. *Journal of clinical psychology in medical settings* 2014; 21: 337-346.

3. Ruehl CA, Landry KK, Stoiber KC, et al. Building a Cardiac Educational Achievement Partnership Program: Examination of Implementation. *Circulation: Cardiovascular Quality and Outcomes* 2022; 15: e008531.

4. Chorna O, Baldwin HS, Neumaier J, et al. Feasibility of a team approach to complex congenital heart defect neurodevelopmental follow-up: early experience of a combined cardiology/neonatal intensive care unit follow-up program. *Circulation: Cardiovascular Quality and Outcomes* 2016; 9: 432-440.

5. Michael M, Scharf R, Letzkus L, et al. Improving neurodevelopmental surveillance and follow‐up in infants with congenital heart disease. *Congenital Heart Disease* 2016; 11: 183-188.

6. Davis JAM, Miller-Tate H and Texter KM. Launching a new strategy for multidisciplinary management of single-ventricle heart defects. *Critical Care Nurse* 2018; 38: 60-71.

7. Loccoh EC, Yu S, Donohue J, et al. Prevalence and risk factors associated with non-attendance in neurodevelopmental follow-up clinic among infants with CHD. *Cardiology in the Young* 2018; 28: 554-560.

8. Di Maria MV, Barrett C, Rafferty C, et al. Initiating a Fontan multidisciplinary clinic: decreasing care variability, improving surveillance, and subsequent treatment of Fontan survivors. *Congenital Heart Disease* 2019; 14: 590-599.

9. Monteiro SA, Serrano F, Tsang R, et al. Ancillary referral patterns in infants after initial assessment in a cardiac developmental outcomes clinic. *Congenital Heart Disease* 2019; 14: 797-802.

10. Glotzbach KL, Ward JJ, Marietta J, et al. The benefits and bias in neurodevelopmental evaluation for children with congenital heart disease. *Pediatric Cardiology* 2020; 41: 327-333.

11. Tan A, Semmel ES, Wolf I, et al. Implementing standard screening for autism spectrum disorder in CHD. *Cardiology in the Young* 2020; 30: 1118-1125.

12. Alam S, Ilardi D, Cadiz E, et al. Impact of Cardiac Neurodevelopmental Evaluation for Children with Congenital Heart Disease. *Developmental Neuropsychology* 2022; 47: 32-41.

13. Favilla E, Faerber JA, Hampton LE, et al. Early evaluation and the effect of socioeconomic factors on neurodevelopment in infants with tetralogy of Fallot. *Pediatric cardiology* 2021; 42: 643-653.

14. Lee TL, Ronai C, Saxton SN, et al. Congenital heart disease and neurodevelopmental evaluation: National guidelines vs. single center utilization. *Progress in Pediatric Cardiology* 2021; 62: 101384.

15. Robertson CM, Sauve RS, Joffe AR, et al. The registry and follow-up of complex pediatric therapies program of Western Canada: a mechanism for service, audit, and research after life-saving therapies for young children. *Cardiology Research and Practice* 2011; 2011.

16. Roberts SD, Kazazian V, Ford MK, et al. The association between parent stress, coping and mental health, and neurodevelopmental outcomes of infants with congenital heart disease. *The Clinical Neuropsychologist* 2021; 35: 948-972.

17. Fourdain S, Caron-Desrochers L, Simard M-N, et al. Impacts of an Interdisciplinary developmental follow-up program on neurodevelopment in congenital heart disease: the CINC Study. *Frontiers in Pediatrics* 2020: 611.

18. Fourdain S, Simard M-N, Dagenais L, et al. Gross motor development of children with congenital heart disease receiving early systematic surveillance and individualized intervention: brief report. *Developmental Neurorehabilitation* 2021; 24: 56-62.

19. Eagleson K, Campbell M, McAlinden B, et al. Congenital Heart Disease Long‐term Improvement in Functional hEalth (CHD LIFE): A partnership programme to improve the long‐term functional health of children with congenital heart disease in Queensland. *Journal of Paediatrics and Child Health* 2020; 56: 1003-1009.

20. Eagleson K, Campbell M, McAlinden B, et al. 659 Early Outcomes of the CHD LIFE Program Long-Term Developmental Care Pathway for High-Risk Children With Congenital Heart Disease. *Heart, Lung and Circulation* 2020; 29: S333-S334.

21. Quadir A, Popat H, Crowle C, et al. Neurodevelopmental outcomes in neonatal extracorporeal membrane oxygenation survivors: An institutional perspective. *Journal of Paediatrics and Child Health* 2022; 58: 1811-1815.

22. Domanski O, Joriot S, Houeijeh A, et al. Initiation of a systematic screening for neurodevelopmental disorder program for infants with congenital heart disease. *Archives of Cardiovascular Diseases Supplements* 2022; 14: 243-undefined. DOI: 10.1016/j.acvdsp.2022.07.048.

23. Domanski O, Joriot S, Houeijeh A, et al. Neurodevelopmental disorder and congenital heart disease: Initiating a systematic follow-up program. *Cardiology in the Young* 2022; 32: S128-undefined. DOI: 10.1017/S1047951122001950.
