# Supplementary material for: Implementing neurodevelopmental follow‐up care for children with congenital heart disease: A scoping review with evidence mapping
Source: Dev Med Child Neurol. 2023 Jul 8;66(2):161–75. doi: 10.1111/dmcn.15698 (PMC10953404; doi:10.1111/dmcn.15698)
Supplement: Supplementary file 9 — Table S5: Detailed descriptions of international practices and overviews of neurodevelopmental follow‐up of children with CHD reported in included surveys. [file DMCN-66-161-s009.docx]

**Table S5.** Detailed descriptions of international practices of neurodevelopmental follow-up of children with CHD reported in included surveys and overviews

| *Author, Year* | *Geographical region* | *Volume of patients* | *Overview of programs/services offered* | *Key providers* | *Frequency or timing of assessment* | *Tools and assessment commonly reported* |
| --- | --- | --- | --- | --- | --- | --- |
| Knutson et al, 2016 ^1^ | USA | 61% of respondents look after less than 10 children with CHD | Almost all physicians and paediatricians surveyed (n= 187, 94%) perform regular general developmental screening in own practice. 49% (n=99) mostly or always referred children with CHD for formal developmental evaluation. | 85% of providers who responded have access to specialists to complete a formal assessment/evaluation. Most (n=187, 93%), have not received guidance from a paediatric cardiologist. | Most (n=143, 71%) provide general developmental screening at all preventive care visits | Not reported |
| Di Maria et al, 2019 ^2^ | USA | Most sites saw 151-300 children per year, range from less than 50 to more than 300 per year | Variety of Fontan follow-up clinics from ‘as needed’ consults to large systematic clinical programs. Providers routinely order neurodevelopment testing in 54% programs, the rest as needed. | Primarily cardiologist, nurses, and advanced practice providers. Psychologist only routinely involved in 3/11 sites. Referral to psychologist available in 7/11 sites. | Every other year (33%), 3-4 year intervals (50%), >5 year intervals (17%) | - Child Behaviour Checklist - Pediatric Quality of Life Inventory™ |
| Miller, et al, 2020 ^3^ | USA | Sites performed 85 to 428 unique evaluations per year (40-120 most common) | Range of cardiac neurodevelopmental follow-up programs | Psychologist, developmental paediatrician, psychiatrist, paediatrician or neurologist responsible for evaluation and assessment | Range of 2-8 visits for children but 3-5 most common. Median of 5 visits for those under 5. 68% of consults per year were for those under 3 years old. | - Bayley Scales of Infant and Toddler Development - Behavior Assessment System for Children-3 - Adaptive Behaviour Assessment System - Delis–Kaplan Executive Function System - California Verbal Learning Test - Wechsler Preschool and Primary Scale of Intelligence - Beery Visual Motor Integration |
| Sidhu et al, 2022 ^4^ | USA | 24% of respondents saw <10 children with CHD per week, 50% saw 11-30, 26% saw >30 children per week | 69% of paediatric cardiologists surveyed had access to a ND follow-up program. Of the remainder, 25% did not refer for ND follow-up, 45% performed ND surveillance and referred to specialist, 30% referred all children with CHD for surveillance. | Developmental paediatrician (35%), paediatric cardiologist (35%) as director of ND programs. If no ND program, generally referred to primary care physician, developmental paediatrician, neurologist or early intervention. | Not reported | Not reported |
| Basile et al, 2022 ^5^ | USA | Hospitals included performed a mean of 365 cardiac procedures at annually (adult and paediatric) | Specialised cardiac neurodevelopmental program available to 32 (67%) of outpatient paediatric cardiac clinics. 56% of cardiac teams do not conduct developmental screening/evaluation while 44% of cardiac clinics (n=21) systematically screen and evaluate for neurodevelopmental issues. | Not reported | 30-40% of all sites screened at 9, 18, and 24-30 months. One-quarter screened at 3-5 years and 12.5 % at 11-12 years | Not reported |
| Kasparian et al, 2022^7^ | USA and Canada | Hospitals/sites included had a median surgical volume of 426 cardiac procedures annually (adult and paediatric). Annual site volumes ranged from 57 to 1306 | Face-to-face neurodevelopmental assessment for infants and children with CHD at 90% of sites. Interventions for children (50% sites), caregivers (43% sites) or families (33% sites).  During the COVID-19 pandemic 24/30 sites provided services using telehealth. These were mostly interviews or interventions with children and caregivers, and discussion of test results. Only 1/23 sites performed standardised infant ND evaluation via telehealth. Most transitioned to parent rating scales or observational methods. | Not reported | Not reported | - Behavior Rating Inventory of Executive Function - Behavior Assessment System for Children-3 - Adaptive Behaviour Assessment System - Modified Checklist for Autism in Toddlers - Social Responsiveness Scale - Ages and Stages Questionnaire - Wechsler Intelligence Scale for Children - Delis–Kaplan Executive Function System - Wechsler Individual Achievement Test - California Verbal Learning Test - Developmental Assessment of Young Children-2 |
| Leon et al, 2022^6^ | USA and Canada | All participating sites were large volume hospitals with >400 annual admissions or >25 NICU beds | Of surveyed sites, 25 (86%) routinely perform ND follow-up, 3 perform it sometimes, and 1 site only has a program for patients with hypoplastic left heart syndrome. Programs are mostly embedded in paediatric cardiology (15/29), the neonatology program (8/29), or speciality complex care programs (3/29). Several sites (3/29) divide care between specialities based on the child’s diagnosis. | Not reported | Not reported | - Bayley Scales of Infant and Toddler Development - Ages and Stages Questionnaire |
| Bolduc et al, 2022 ^12^ | Canada | Sites performed between 69 and 900 surgeries per year. Median of 108 surgeries annually. | Four types of models observed across 8 sites. (1) 4 sites (50%) had structured programs for developmental follow-up surveillance, screening, and evaluation, (2) one site systematically referred for psychological evaluation, (3) one site used a general neonatal follow-up program with developmental paediatricians, (4) 2 sites (25%) had no formal programs or surveillance. |  | Most often twice during the first year of life, then every 1-2 years until 5 years old. Psychology assessment model just at 18-24 months and 5 years | Mostly in-person screening and evaluation tools. Some phone and web-based questionnaires. Parental screening of mental health at 25% of centres. Wide range of tools used:   - Bayley Scales of Infant and Toddler Development - Ages and Stages Questionnaire - Child Behaviour Checklist - Behaviour Assessment System for Children-3 - Adaptive Behaviour Assessment System - Behaviour Related Inventory of Executive Functioning - California Verbal Learning Test - Wechsler Preschool and Primary Scale of Intelligence - Abnormal Involuntary Movement Scale\ - Beery Visual Motor Integration - Modified Checklist for Autism in Toddlers - Parenting Stress Index Short Form |
| Hoskote et al, 2021 ^8^ | UK | Not reported | Reviewed frequency of use of four types of service categories: (1) 21% accessed child development, neurology or general paediatrics (2), 17% accessed special senses (e.g., audiology, ophthalmology), (3) 19% had accessed tertiary specialists not linked to child development, (4) 9% had accessed dietetics. | Not reported | Not reported | Not reported |
| Smith et al, 2019 ^11^ | South Africa | Half of those surveyed cared for >25 children with CHD. 20% care for > 50 children with CHD. | 75% of paediatricians and cardiologists surveyed (n=15) routinely performed developmental surveillance in own practice. 65% (n=13) routinely referred children with CHD for developmental screening. | Paediatrician, cardiologist or occupational therapist in first instance. Then referred to allied health services (75%) or paediatric neurologists/ neurodevelopmental paediatricians (55%), for formal developmental evaluation. | Most (75%) providers referred children for developmental screening at any care visit (not fixed ages) | - Bayley Scales of Infant and Toddler Development - Abnormal Involuntary Movement Scale - Delis–Kaplan Executive Function System |
| Feldmann et al, 2022 ^9^ | Europe | Surveyed sites performed from <100 to >250 paediatric cardiac surgeries per year. One-third performed >250 per year | 10 (40%) of paediatric cardiac sites surveyed had a structured follow-up neurodevelopmental program, 24% had plans to commence a program | Not reported | Not reported | Not reported |
| Natterer et al, 2022^10^ | Switzerland | 80-120 neonates with CHD are operated on in first 6 weeks of life annually in Switzerland | Follow-up for children with CHD integrated into local follow-up programs for all high-risk infants (e.g. pre-term). Referred to centre closet to home before discharge. At all follow-up timepoints: medical history, socioeconomic history, physical exam and somatic growth, neurodevelopmental assessment. | Not reported | At 9-12 months, 18-24 months, and 5.5-6 years. | - Bayley Scales of Infant and Toddler Development (0-42 months) - Kaufman Assessment Battery for Children (from 5.5 years) |

CHD, congenital heart disease; USA, United States of America; ND, neurodevelopmental; UK, United Kingdom

# References

1. Knutson S, Kelleman MS and Kochilas L. Implementation of developmental screening guidelines for children with congenital heart disease. *The Journal of Pediatrics* 2016; 176: 135-141. e132.

2. Di Maria MV, Brown DW, Cetta F, et al. Surveillance testing and preventive care after Fontan operation: a multi-institutional survey. *Pediatric cardiology* 2019; 40: 110-115.

3. Miller TA, Sadhwani A, Sanz J, et al. Variations in practice in cardiac neurodevelopmental follow-up programs. *Cardiology in the Young* 2020; 30: 1603-1608.

4. Sidhu SK, McLaughlin LJ, Pham TB, et al. Paediatric cardiologist adherence to American Heart Association neurodevelopmental recommendations for CHD patients. *Cardiology in the Young* 2022: 1-7.

5. Basile NL, Kirschman KJB and Dempster NR. Psychosocial, neurodevelopmental, and transition of care practices provided to children with CHD across North American cardiac clinics. *Cardiology in the Young* 2022: 1-7.

6. Leon RL, Levy PT, Hu J, et al. Practice variations for fetal and neonatal congenital heart disease within the Children’s Hospitals Neonatal Consortium. *Pediatric Research* 2022: 1-8.

7. Kasparian NA, Sadhwani A, Sananes R, et al. Telehealth Services for Cardiac Neurodevelopmental Care during the COVID-19 Pandemic: A Site Survey from the Cardiac Neurodevelopmental Outcome Collaborative. *Cardiology in the Young* 2022. DOI: 10.1017/S1047951122000579.

8. Hoskote A, Ridout D, Banks V, et al. Neurodevelopmental status and follow-up in preschool children with heart disease in London, UK. *Archives of Disease in Childhood* 2021; 106: 263-271.

9. Feldmann M, Hagmann C, de Vries L, et al. Neuromonitoring, neuroimaging, and neurodevelopmental follow-up practices in neonatal congenital heart disease: a European survey. *Pediatric Research* 2022: 1-8.

10. Natterer J, Schneider J, Sekarski N, et al. ORCHID (Outcome Registry for CHIldren with severe congenital heart Disease) a Swiss, nationwide, prospective, population-based, neurodevelopmental paediatric patient registry: framework, regulations and implementation. *Swiss medical weekly* 2022; 152: w30217-undefined. DOI: 10.4414/smw.2022.w30217.

11. Smith R, Le Roux H, Nel H, et al. Neurodevelopmental evaluation and referral practices in children with congenital heart disease in central South Africa. *SA Heart* 2019; 16: 324-332.

12. Bolduc M-E, Rennick JE, Gagnon I, et al. Canadian developmental follow-up practices in children with congenital heart defects: a national environmental scan. *CJC Pediatric and Congenital Heart Disease* 2022; 1: 3-10.
